# Tocopherol deficiency reduces sucrose export from salt-stressed potato leaves independently of oxidative stress and symplastic obstruction by callose

**DOI:** 10.1093/jxb/eru453

**Published:** 2014-11-26

**Authors:** María Amparo Asensi-Fabado, Alexandra Ammon, Uwe Sonnewald, Sergi Munné-Bosch, Lars M. Voll

**Affiliations:** ^1^University of Barcelona, Faculty of Biology, Department of Plant Biology, Diagonal Avenue 643, E-08028 Barcelona, Spain; ^2^Friedrich-Alexander-University Erlangen-Nuremberg, Division of Biochemistry, Staudtstr. 5, D-91058 Erlangen, Germany

**Keywords:** Oxidative stress, potato, salt stress, SnRK1 signalling, starch accumulation, sucrose export defective, tocopherol, tuber yield.

## Abstract

Tocopherol cyclase, encoded by the gene *SUCROSE EXPORT DEFECTIVE1*, catalyses the second step in the synthesis of the antioxidant tocopherol. Depletion of SXD1 activity in maize and potato leaves leads to tocopherol deficiency and a ‘sugar export block’ phenotype that comprises massive starch accumulation and obstruction of plasmodesmata in paraveinal tissue by callose. We grew two transgenic StSXD1:RNAi potato lines with severe tocopherol deficiency under moderate light conditions and subjected them to salt stress. After three weeks of salt exposure, we observed a strongly reduced sugar exudation rate and a lack of starch mobilization in leaves of salt-stressed transgenic plants, but not in wild-type plants. However, callose accumulation in the vasculature declined upon salt stress in all genotypes, indicating that callose plugging of plasmodesmata was not the sole cause of the sugar export block phenotype in tocopherol-deficient leaves. Based on comprehensive gene expression analyses, we propose that enhanced responsiveness of SnRK1 target genes in mesophyll cells and altered redox regulation of phloem loading by SUT1 contribute to the attenuation of sucrose export from salt-stressed SXD:RNAi source leaves. Furthermore, we could not find any indication that elevated oxidative stress may have served as a trigger for the salt-induced carbohydrate phenotype of SXD1:RNAi transgenic plants. In leaves of the SXD1:RNAi plants, sodium accumulation was diminished, while proline accumulation and pools of soluble antioxidants were increased. As supported by phytohormone contents, these differences seem to increase longevity and prevent senescence of SXD:RNAi leaves under salt stress.

## Highlight

Analysis of salt-challenged, tocopherol-deficient potato plants revealed that stress-induced blocking of sugar export is not caused by plasmodesmatal plugging, but rather by direct effects of sugar signalling on export.

## Introduction

Tocopherols are amphiphilic antioxidants exclusively synthesized by photosynthetic organisms and are present in all plant organs (Asensi-Fabado and Munné-Bosch, 2010). They are composed of a hydrophobic prenyl side chain and a polar chromanol ring system that is differently methylated in α-, ß-, γ-, and δ-tocopherol. The four tocopherol species differ in the degree and position of these methyl groups at the chromanol ring. Together with tocotrienols, tocopherols are collectively known as tocochromanols or vitamin E. Tocopherols are considered to play an essential antioxidant role in plants based on their excellent antioxidant activity *in vitro*, which is conferred by the capacity of the chromanol ring to donate a phenolic hydrogen to lipid peroxyl radicals (Liebler, 1993; Kamal-Eldin and Appelqvist, 1996). Thus, an important function of tocopherols in photosynthetic organisms is protection from lipid peroxidation (Sattler *et al.*, 2004; Maeda *et al.*, 2005). Furthermore, tocopherols can quench and scavenge singlet oxygen, which is a threat under photo-oxidative stress conditions (Trebst *et al.*, 2002; Munné-Bosch *et al.*, 2005; Kobayashi and DellaPenna, 2008). Due to their antioxidant activity and membrane-stabilizing properties (Hincha, 2008), tocopherols are considered to play a major role in the protection of the photosynthetic apparatus.

Since the isolation of the *vte1* mutant by Porfirova *et al.* (2002), functional studies in *Arabidopsis thaliana* have led to a better understanding of tocopherol function. The *vte1* mutant is deficient in the second step of tocopherol biosynthesis, involving tocopherol cyclase (TC), and is completely devoid of tocopherols, but still accumulates the amphiphilic precursor 2,2-dimethyl-5-phytyl-benzoquinone (DMPBQ; Porfirova *et al.*, 2002; Sattler *et al.*, 2003). Exposure of the *vte1* mutant to high light slightly reduced its chlorophyll content and photosynthetic quantum yield in comparison to the wild type (Porfirova *et al.*, 2002). A later study has shown that compensatory mechanisms, such as an increase in non-photochemical quenching, contribute to the prevention of excess photooxidative damage in the *vte1* mutant under high light conditions (Havaux *et al.*, 2005). The *Arabidopsis* homogentisate phytyltransferase (HPT) mutant *vte2* is devoid of the committed step in tocopherol biosynthesis, the prenylation of homogentisic acid by phytyl diphosphate, and does not accumulate any intermediates like DMPBQ (Sattler *et al.*, 2003, 2004). The *Arabidopsis vte2* mutant showed impaired seed longevity and seedling growth during germination, as well as increased expression of many defence-related genes (Sattler *et al*., 2004, 2006), while tocopherol-deficient transgenic tobacco plants silenced for HPT exhibited accelerated senescence (Abbasi *et al.*, 2009). All three effects can be connected to the antioxidant role of tocopherols.

A striking effect of tocopherol deficiency is the accumulation of soluble sugars and starch in source leaves of the maize TC mutant *sxd1* (*sucrose export defective1*; Russin *et al.*, 1996; Botha *et al.*, 2000) and transgenic StSXD1:RNAi-silenced potato plants (Hofius *et al.*, 2004), which is caused by callose occlusion of plasmodesmata in phloem-associated cells in both systems. The link between photoassimilate export and tocopherol function is still unclear, given that tocopherol-deficient *Arabidopsis* (Maeda *et al.*, 2006) and tobacco (Abbasi *et al.*, 2009) plants did not exhibit this sugar export block phenotype under normal growth conditions. Among a range of abiotic stresses, only cold stress was able to trigger sugar and starch accumulation as well as callose deposition in the phloem tissue of *vte2* source leaves, and to a lesser extent in *vte1* source leaves (Maeda *et al.*, 2006). Altered polyunsaturated fatty acid (PUFA) metabolism in the endoplasmic reticulum rather than photoinhibition and lipid peroxidation accounted for this cold stress-inducible sugar export block in *vte2* mutants (Maeda *et al.*, 2008). These results suggest that tocopherols can influence extra-plastidial processes independent of their antioxidant function, which supports a role for tocopherols in intracellular signalling (Provencher *et al.*, 2001; Munné-Bosch and Falk, 2004). In line with this, studies on animals have proven that tocopherols can modulate membrane-associated signalling and gene expression (Azzi *et al.*, 2002; Brigelius-Flohé *et al.*, 2002). More recently, it has been found that accumulation of γ- instead of α-tocopherol in the *vte4* mutant of *Arabidopsis* influences nuclear gene expression of the ethylene signalling pathway (Cela *et al.*, 2011).

Only a little is known about the role of tocopherols in salt stress. Tocopherol-depleted HPT:RNAi tobacco plants showed a decreased tolerance to salt stress and enhanced lipid peroxidation upon salt challenge (Abbasi *et al.*, 2007). However, HPT-silenced tobacco did not exhibit a sugar export block either under control or salt-stress conditions. Here, we used two tocopherol-deficient SXD1:RNAi transgenic potato lines silenced for TC that were generated in a previous study and are known to exhibit a sugar export block when grown at a moderate PFD of 500 µE m^–2^ s^–1^ (Hofius *et al.*, 2004). We grew these plants at a lower PFD of 250 μE m^–2^ s^–1^, in which no sugar export block occurs. To investigate the connection between oxidative and ionic stress caused by salt treatment, tocopherol deficiency, and carbohydrate export, we challenged SXD1:RNAi plants with salt stress by watering with 150mM NaCl. We found impaired sugar export from source leaves in salt-stressed SXD1:RNAi potato plants, which resulted in a stronger reduction of tuber yield in the transgenic plants compared to the wild type. However, no excessive callose deposition in the phloem could be observed in salt-stressed transgenic plants. Moreover, these plants did not show higher oxidative stress or lipid peroxidation than wild-type plants, but a reduced salt uptake that was probably caused by diminished photosynthetic gas exchange compared to the wild type. Based on our data, we present a model explaining how reduced sugar export is caused in tocopherol-deficient SXD:RNAi potato upon salt stress.

## Materials and methods

### Plant material, growth conditions, and sampling

*Solanum tuberosum* L. var. Solara (potato) was obtained from Bioplant (Ebstorf, Germany). Tocopherol-deficient transgenic lines StSXD1:RNAi-21 and -22 had been generated in a previous study (Hofius *et al.*, 2004) by constitutively expressing an intron-spliced hairpin RNA (RNAi) construct targeted at *StSXD1*, which encodes TC.

All potato plantlets used for our experiments were vegetatively propagated from stem cuttings in tissue culture under a 16-h light/8-h dark regime (150 μmol m^–2^ s^–1^) at 21°C and 50% relative humidity on Murashige and Skoog medium (Sigma, St Louis, USA) containing 2% (w/v) sucrose. Rooted plantlets were subsequently grown in the greenhouse in individual 4-l pots at 50% humidity, with supplementary light (250 μmol m^–2^ s^–1^) under a 16-h day/8-h night (22°C/18°C) regime. The salt treatment started 38 days after transfer to soil, and consisted of irrigation with tap water without (control plants) and with additional salt (150mM NaCl). Plants were irrigated with 200ml of the corresponding solution every other day for the first 10 days of treatment. From day 11 to day 39, control plants received 250ml daily water application until they reached maturity at day 39 after the onset of treatment. To avoid waterlogging, salt-treated plants were irrigated daily with 125ml saline solution (150mM NaCl) from day 11 to day 20, and irrigated every other day with 125ml 150mM NaCl from day 21 to day 39. At 39 days after the onset of treatment, salt application was stopped and salt-treated plants were irrigated further with 100ml tap water every other day until the harvest of tubers at day 58. At the time of harvest, NaCl accumulation in the soil was comparable for pots with wild-type Solara (17.6±1.2mg NaCl mg^–1^ soil dry weight), SXD21:RNAi (19.5±1.3mg NaCl mg^–1^ soil dry weight), and SXD22:RNAi (17.6±2.1mg NaCl mg^–1^ soil dry weight).

After 2 weeks of treatment, a reduction in plant size was evident in salt-treated plants compared to their respective controls. Therefore, leaf gas exchange rate and PSII quantum efficiency were assessed as described below. At 19 days of treatment, before the appearance of senescence in salt-treated plants, fully illuminated leaf samples were collected at three whorl positions to perform physiological measurements: top (young leaves, leaf 3), middle (fully expanded leaves, leaf 8), and bottom (leaf 11, just above the lowest leaf). At the time of sampling, the leaf water potential of salt-stressed middle and bottom leaves was comparable between all genotypes (Supplementary Figure S3). While leaf water potential was not substantially increased upon salt stress in middle and bottom leaves, salt-stressed top leaves exhibited a 5-fold increase in water potential compared to the controls. Leaves for biochemical analyses were sampled towards the end of the photoperiod (12h into the light period), snap frozen in liquid nitrogen and kept at –80°C until measurement. In addition, middle leaves were collected at the end of the subsequent night period for sugar and starch measurements. Aerial and tuber biomass were determined at 20 days of treatment with one subset of plants, while tuber biomass was measured again at the end of the plant life cycle, 58 days after the onset of treatment.

### Quantification of tocopherol, soluble sugars, starch, and free amino acids

Tocopherol, soluble sugar, starch, and free amino acid contents were determined from aliquots of 20–30mg leaf tissue as described in Abbasi *et al.* (2007). While tocopherol and amino acid contents were determined after HPLC separation by fluorescence detection, soluble sugars and starch were quantified using a spectrophotometric assay.

### Determination of malondialdehyde, ascorbate, and glutathione levels

Malondialdehyde (MDA) and ascorbate were extracted and quantified spectrophotometrically as described in detail by Abbasi *et al.* (2007). Glutathione was determined by reversed-phase HPLC following the protocol of Abbasi *et al.* (2009). For ascorbate and MDA measurements, 50mg leaf tissue was used per sample; for glutathione measurement, 30mg leaf tissue was used per sample.

### Quantification of intermediates of central carbohydrate and carboxylate metabolism

Phosphorylated intermediates and major carboxylic acids were determined by IC-MS/MS of perchloric acid extracts of 50–100mg leaf tissue as described by Horst *et al.* (2010).

### Measurement of invertase activity

Invertase activity was determined according to the spectrophotometric assay described in Horst *et al.* (2008).

### Quantification and histochemical localization of callose

Quantification of leaf callose content was performed as described (Köhle *et al.*, 1985), using a SpectraMax Gemini XS spectrofluorometer (Molecular Devices, Ismaning, Germany). Callose content was expressed as β-1,3-glucan pachyman equivalents.

For histochemical localization of callose, whole potato leaflets were preserved in 80% ethanol after collection. Leaflets were destained by incubation in 80% ethanol at 80ºC for 30min, followed by 30min dark incubation in 0.07M sodium phosphate buffer, pH 9, then 60min incubation in 0.005% aniline blue solution. After rinsing with water, samples were observed by fluorescence microscopy (excitation filter, BP 340–380nm; dichromatic mirror, 400nm; suppression filter, LP 340nm) with a Leica DMR microscope (Leica Microsystems GmbH, Wetzlar, Germany). Images were taken with a Spot Flex CCD camera (Diagnostic Instruments, Sterling Heights, MI, USA).

### Measurement of leaf sugar exudation rate

Fully expanded source leaves (leaf 9) were excised at the base of the petiole 5h after the onset of the light period. Submerged petioles were recut and subsequently placed in 15ml 20mM EDTA solution, while leaves were kept at growth light conditions. The exudate from the first 30min was discarded. Then, the collection tube was changed at 1h intervals for assessment of changes in exudation rate over a time course of 3h. Sucrose concentration in the exudates was determined spectrophotometrically as described by Abbasi *et al.* (2009). Sugar exudation rate was calculated on a leaf area basis after correcting for differences in transpiration between the sampled leaves.

### Gas-exchange and photosynthetic performance measurements

Photosynthetic parameters (A, E, ETR, and Fv/Fm) were determined at a PFD of 400 μmol m^–2^ s^–1^ with a combined gas exchange/chlorophyll imaging system (GFS-3000 and MINI-Imaging-PAM chlorophyll fluorometer, Walz, Effeltrich, Germany) at 350 ppm CO_2_, 13 000 ppm H_2_O, and a leaf temperature of 22°C as described by Horst *et al.* (2008).

### Elemental analysis

Leaf samples were oven dried, and 50mg dry tissue was acid digested with 1ml 70% HNO_3_ and 0.5ml 30% H_2_O_2_ (Baker Instra grade) in closed Teflon vessels at 90°C overnight. Samples were then mixed with 20ml ultrapure H_2_O, and sodium (Na), potassium (K), and calcium (Ca) were determined by Inductively Coupled Plasma Optical Emission Spectrometry (ICP-OES) with a Perkin Elmer Optima 3200RL spectrometer (Waltham, USA). Sample solutions showing an Na concentration below the ICP-OES detection limit (3 ppm) were also measured by atomic absorption spectrometry using a Varian AA240FS spectrometer (Palo Alto, USA).

### Measurement of leaf osmolality

Potato leaf discs of 0.6cm^2^ were homogenized and, after centrifugation at 14 000rpm for 3min, 5 to 10 μl supernatant were mixed with ultrapure H_2_O up to a final volume of 100 μl. Solutions were measured using a freezing-point micro-osmometer (Vogel OM815, Giessen, Germany).

### Hormonal profiling

Levels of ABA (abscisic acid), ACC (the ethylene precursor, 1-amino-cyclopropane-1-carboxylic acid), SA (salicylic acid), JA (jasmonic acid), IAA (indole-3-acetic acid), IPA (isopentenyladenosine), 2-IP (isopentenyladenine), Z (zeatin), ZR (zeatin riboside), DHZ (dihydrozeatin), and DHZR (dihydrozeatin riboside) were simultaneously analysed by UPLC-ESI/MS/MS using deuterium-labelled hormone analogues as internal standards as described by Müller and Munné-Bosch (2011). In short, leaf samples (50mg) were extracted in a final volume of 400 μl methanol:isopropanol:glacial acetic acid mixture, 40:59:1 (v/v/v), including a re-extraction. After filtration through a 0.2 µm PTFE filter (Waters, Milford, MA, USA), fresh extracts were injected into the UPLC–ESI/MS/MS system. Chromatography was performed using an Acquity UPLC System (Waters, Milford, MA, USA) with a HALO C18 (Advanced Materials Technology, Inc., Wilmington, USA) column (2.1×75mm, 2.7 µm). ESI/MS/MS detection was carried out using an API 3000 triple quadrupole mass spectrometer (PE Sciex, Concord, Ontario, Canada).

### Gene expression analysis

Transcript amounts of the SnRK1 target genes *ASN1*, *SnRKα*, *SnRKγ, SuSy2*, *SEN1, TPS11*, and *UGE* were determined by qRT-PCR exactly as described by Debast *et al.* (2011). Primers used for transcript quantitation of *StSUT1* (Riesmeier *et al.*, 1993) were qStSUT1fw 5ʹ-TGT CTG GGC AAA TGC TTT GTA-3ʹ and qStSUT1rev 5ʹ-TTC TAC CAA CCC AAA GTA CC-3ʹ. The primers for qRT-PCR quantitation of the *StSUT1* interactors *StSnakin1* and *StPDI* (Krügel *et al.*, 2012; Krügel and Kühn, 2013) were qStSnakin1fw 5ʹ-GTG ATT CAA AGT GCA AGC TGA GAT G-3ʹ, qStSnakin1rev 5ʹ- GTC CCT ATA ACA AGG ACA TTC ATG-3ʹ. qStPDIfw 5ʹ-GCA AAC CTT GAT GCC GAT AA-3ʹ and qStPDIrev 5ʹ-TCT CGG CCA CCA TCA TAA TC-3ʹ. The potato homologue of the *Arabidopsis XTH5* gene was quantified with the primers qStXTH5fw 5ʹ-GGA CCC ATT GGA ACA AGT TGT AAA C-3ʹ and qStXTH5rev 5ʹ-GCCCTGAATCTTTTCATGGCCATT-3ʹ, while the closest homologue of *Arabidopsis* vacuolar proton-coupled pyrophosphatase AtPVP1 was assessed with the primers qStPVP1fw 5ʹ-GGA TTT GCT ATT GGT TCT GCT GCA-3ʹ and qStPVP1rev 5ʹ-CCG ACT AGC AAA CCA ATG AAG ACT-3ʹ. In all cases, potato ubiquitin was used as an internal reference gene, as described by Debast *et al.* (2011).

## Results

### Tocopherol-deficient potato source leaves exhibit sugar export deficiency and impaired nocturnal starch mobilization under salt stress

Knockdown of TC by constitutive expression of an RNAi construct targeted at the *StSXD1* TC gene resulted in tocopherol-deficient potato lines (Hofius *et al.*, 2004). Among the transgenic lines analysed in our previous study (Hofius *et al.*, 2004), StSXD1:RNAi-22, -27, and -21 showed the strongest reduction in foliar tocopherol levels, containing 0.7, 2.1, and 4.1% of wild-type α-tocopherol, respectively. At a PFD of 500 µmol quanta m^–2^ s^–1^, only StSXD1:RNAi-22 and -27, with <2.1% of wild-type α-tocopherol, displayed a clear sucrose export-deficient phenotype (Hofius *et al.*, 2004).

We first assessed whether the reduction in tocopherol content of StSXD1:RNAi-21 and -22 was still comparable to the previous study after nine years of vegetative propagation of the lines on axenic media. In the present study, young three-week-old plantlets of StSXD1:RNAi-22 and -21 exhibited 1.2±0.2% and 1.1±0.2% of wild type α-tocopherol content, respectively, and therefore these two lines were selected for further experiments.

**Fig. 1.**
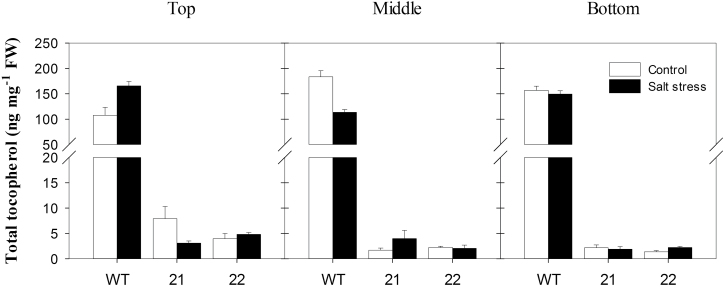
Total tocopherol content of *StSXD1*-silenced potato leaves. Total toopherol levels were calculated on a fresh weight (FW) basis, for StSXD1:RNAi-21 and -22 potato lines and the wild type (WT), at top (leaf 3, left panel), middle (leaf 8, middle panel), and bottom (leaf 11, right panel) whorl positions. Samples were collected 19 days after the onset of treatments, consisting of irrigation either with 150mM NaCl (salt stress, black bars) or water (control, white bars). Data represent the mean ± SE of four individual plants.

Tocopherol content in stressed and control leaves at different whorl positions was further analysed in 57-day-old plants, 19 days after the onset of salt treatment (Fig. 1). Total tocopherol was determined as the sum of α-, γ- and δ-tocopherol (β-tocopherol was below the detection limit). α-tocopherol accounted for 95–98% of total tocopherol content in wild-type leaves, and for >99% of the total tocopherol in leaves of transgenic plants. In control conditions, the reduction in tocopherol content in middle and bottom leaves of both transgenic lines was similar to that of the initially screened plantlets (see Materials and Methods). Overall, tocopherol contents did not change significantly upon salt stress in all genotypes. However, salt-stressed bottom leaves of the transgenic plants exhibited the strongest tocopherol deficiency (1.3% of the wild type in line 21 and 1.5% of the wild type in line 22).

**Fig. 2.**
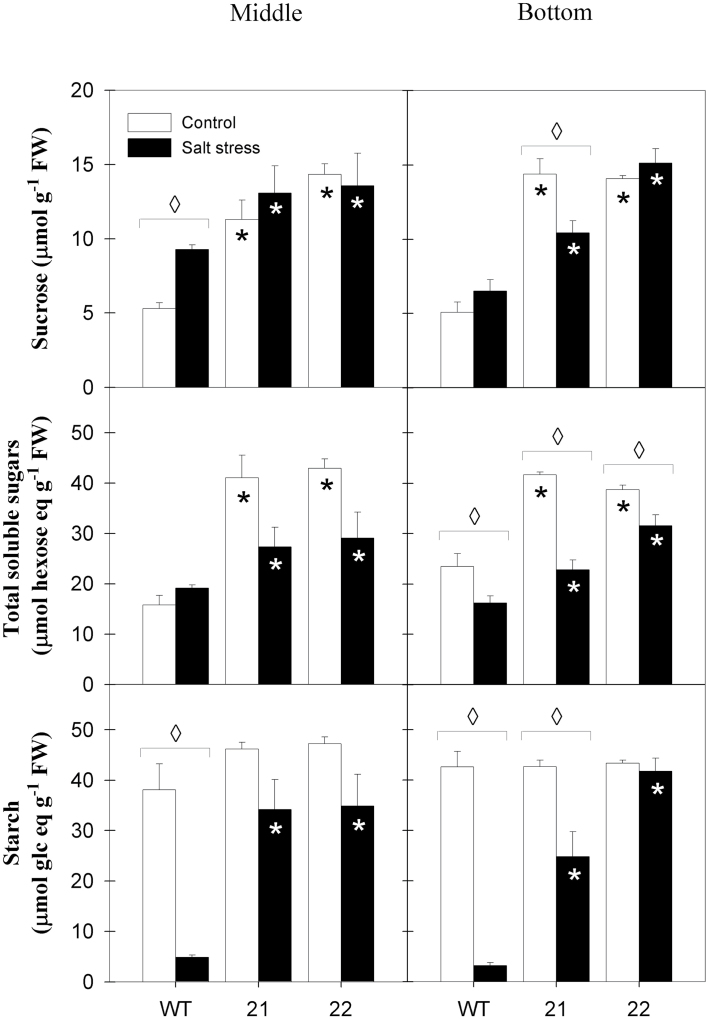
Carbohydrate levels in source leaves of tocopherol-deficient potato plants upon salt treatment. Data represent sucrose, total soluble sugars (glucose, fructose, and sucrose) expressed as hexose equivalents and starch contents (expressed as glucose equivalents) of middle (leaf 8, left panel) and bottom (leaf 11, right panel) leaves of StSXD1:RNAi-21 and -22 potato lines after 19 days’ treatment (salt stress, black bars; control, white bars). at the end of the light period. Samples were collected 12h after the onset of light. Data represent the mean ± SE of four individual plants. Data were analysed by *t*-test; significant differences between the transgenic lines and the wild type (WT) within a treatment are indicated by a black asterisk (control treatment) or a white asterisk (stress treatment), while diamonds indicate significant differences between control and salt stress within a genotype (*P* < 0.05).

Previously, it was demonstrated that StSXD1-silenced potato lines with less than ~2% residual tocopherol content showed an impaired photoassimilate export when grown at a PFD of 500 µmol quanta m^–2^ s^–1^, leading to a strong accumulation of sugars and starch in source leaves (Hofius *et al.*, 2004). In the present study, plants were grown at a lower PFD of 250 µmol quanta m^–2^ s^–1^. Compared to the previous study, a less pronounced accumulation of total soluble sugars was observed in fully expanded source leaves of both tocopherol-deficient plant lines in control conditions at the end of the light period (Fig. 2). In contrast, we could not observe a significant accumulation of starch in the transgenic plants under control conditions compared to the wild type (Fig. 2). In fully expanded control leaves of SXD:RNAi plants, accumulation of soluble sugars was increased almost 3-fold compared to wild-type control leaves, while bottom leaves of transgenic plants showed a 1.6 to 1.8-fold increase in soluble sugars compared to the wild type in lines 22 and 21, respectively. Starch levels in unstressed bottom leaves were similar between transgenic and wild-type plants.

Salt stress provoked a strong reduction in starch content of both middle and bottom wild-type leaves to 8–12% of the levels observed in control conditions (Fig. 2). In contrast, the starch content of tocopherol-deficient plants did not decrease by >20% upon salt stress. As a result, starch content was increased 7- to 13-fold in middle and bottom leaves of transgenic potato plants compared to stressed wild-type leaves of the respective whorl position. In salt-stressed middle leaves of the wild type, sucrose content was increased by 75% compared to control conditions, while sucrose content was comparable in the transgenic plants in stress and control conditions. Unlike the wild type, tocopherol-deficient plants showed a decrease in total soluble sugar content upon salt stress, (Fig. 2, middle panel). Taken together, source leaves of transgenic plants retained higher soluble sugar levels than the wild type under salt stress, but the differences to the wild type were smaller in stress than in control conditions.

**Fig. 3.**
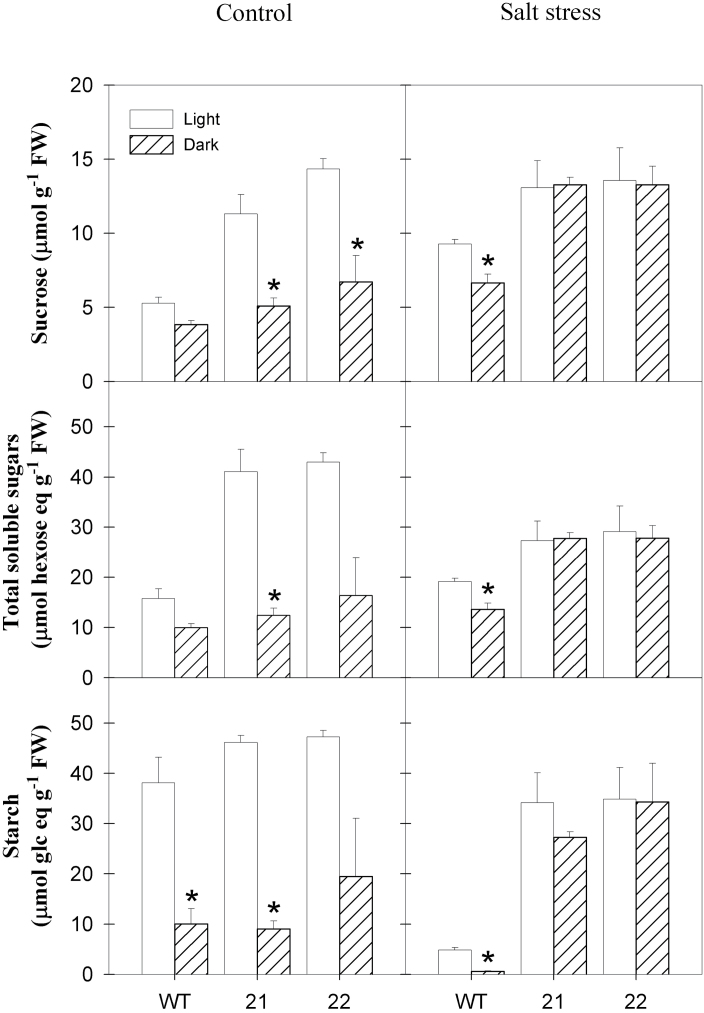
Diurnal carbohydrate turnover in source leaves of *StSXD1*-silenced potato plants challenged with salt stress. Sucrose, total soluble sugars (sum of glucose, fructose, and sucrose) expressed as hexose equivalents and starch contents (expressed as glucose equivalents) were measured in middle leaves (leaf 8) at the end of the light (white bars) and dark (black bars) period. Light samples were collected as stated in the legend to Fig. 2; dark samples were collected immediately before the end of the subsequent dark period. Left panels, control plants; right panels, salt-treated plants; WT, wild type. Data represent the mean ± SE of four individual plants. Data were analysed by *t*-test and significant differences between light and dark period within a genotype are indicated by an asterisk (*P* < 0.05).

In order to clarify whether the transitory starch pool in stressed transgenic plants is not mobilized in the dark due to a sugar export block or whether starch synthesis during the light period remains high in the transgenic plants, middle source leaves were analysed at the end of the dark period (Fig. 3). In control conditions, the turnover of starch was similar between the genotypes, but soluble sugars showed a higher diurnal turnover in tocopherol-deficient plants than in the wild type, suggesting that sugar export was hampered in the transgenic plants during the light phase. In the salt-stressed wild type, the soluble sugar content declined by a quarter during the dark period, which was similar to control conditions. In contrast, soluble sugar content remained constant between the two time points in stressed leaves of the two transgenic lines. Similarly, nocturnal starch mobilization was virtually absent in stressed SXD1:RNAi leaves, while it was elevated in stressed wild-type plants compared to control conditions (87% starch turnover vs 69% starch turnover in control and stress conditions, respectively). Taken together, salt stress was able to reduce soluble sugar accumulation in SXD1-RNAi source leaves, while nocturnal starch mobilization was apparently abolished in the transgenic plants.

### Salt stress does not cause callose accumulation in the phloem, but leads to decreased sugar export and diminished tuber yield in tocopherol-deficient potato plants

**Fig. 4.**
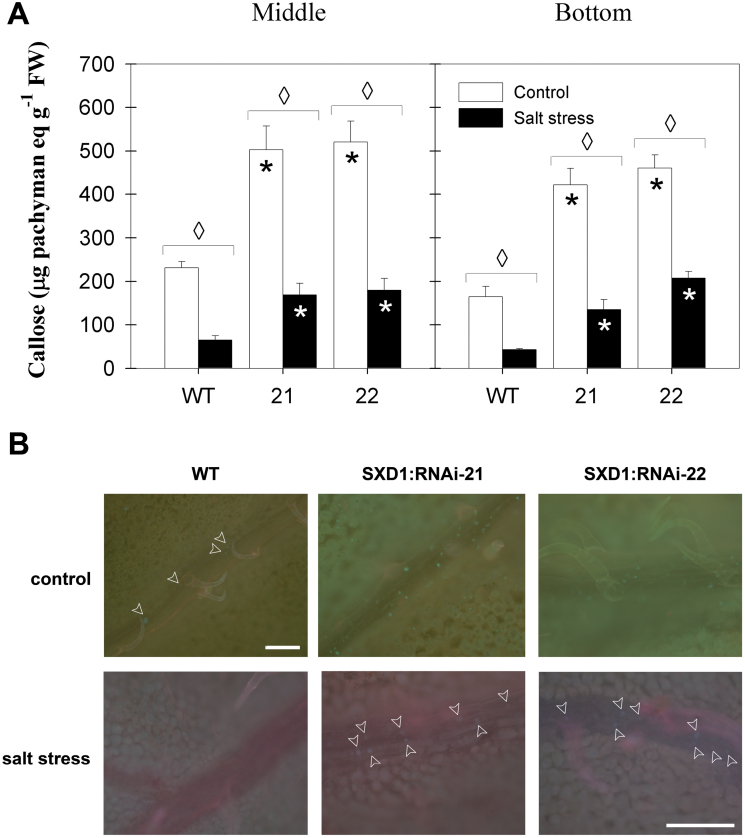
Callose content and tissue distribution in source leaves of *StSXD1*-silenced potato plants 19 days after the onset of salt treatment. (A) Callose content in middle (leaf 8, left panel) and bottom (leaf 11, right panel) leaves of control (white bars) and salt-treated (black bars) plants. Data are given as β-1,3-glucan pachyman equivalents and represent the mean ± SE of four individual plants. Data were analysed by *t*-test; significant differences between the transgenic lines and the wild type (WT) within a treatment are indicated by a black asterisk (control treatment) or a white asterisk (stress treatment), while diamonds indicate significant differences between control and salt stress within a genotype (*P* < 0.05). (B) Fluorescence microscope images of potato middle leaves stained with aniline blue, showing class II and III veins and surrounding mesophyll in the wild type (WT) (left images), SXD:RNAi-21 line (middle images), and SXD:RNAi-22 line (right images). Upper images, control treatment; lower images, salt-stress treatment. Callose was observed in both tocopherol-deficient potato lines as bright spots along the phloem, mainly in control conditions. Arrowheads indicating callose appositions have been included in some images for the sake of clarity. Images are representative of four individual plants per genotype and treatment. The bar in the bottom right image represents 100 μm for all panels except for the top left image, which bears its own reference bar of 100 µm.

The apparent absence of nocturnal starch mobilization in tocopherol-deficient source leaves indicates that major carbohydrate metabolism is disturbed upon salt stress. As outlined in the introduction, previous studies have commonly found that sugar export deficiency of tocopherol-depleted source leaves was a consequence of callose deposition in the vascular tissue, which prevented sucrose loading into the phloem. We therefore analysed whether this was also the case in salt-stressed SXD:RNAi potato plants. Quantitative measurements of callose content in fully expanded and bottom source leaves showed that callose levels in both StSXD1:RNAi lines were increased ~2-fold compared to the wild type plants in control conditions (Fig. 4A). Salt stress caused a marked decrease in callose content in all three genotypes. Microscopic observation of aniline blue-stained leaves confirmed that callose deposition was mainly located in the vasculature of tocopherol-deficient control leaves, while it was barely detectable in wild-type control leaves (Fig. 4B). In agreement with the quantitative results, microscopic analysis showed that callose deposition in salt-stressed, tocopherol-deficient plants was less prominent compared to control conditions.

**Fig. 5.**
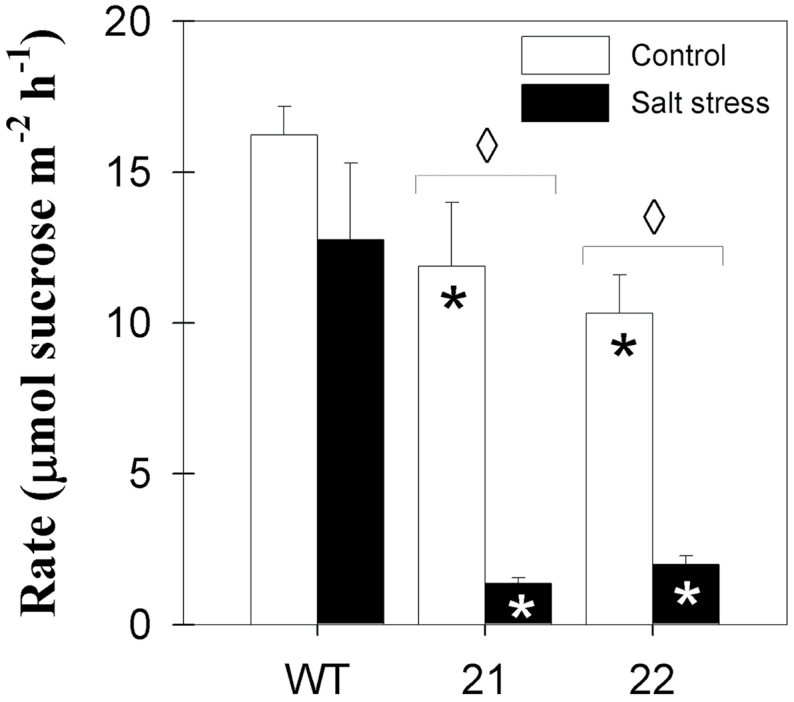
Changes in sucrose exudation rate of source leaves upon salt treatment. Samples were taken from middle leaves (leaf 9) and were collected 19 days after the onset of treatments (salt stress, black bars; control, white bars). Data represent the mean ± SE of four individual plants. Data were analysed by *t*-test; significant differences between the transgenic lines and the wild type within a treatment are indicated by a black asterisk (control treatment) or a white asterisk (stress treatment), while diamonds indicate significant differences between control and salt stress within a genotype (*P* < 0.05). WT, wild tye.

Therefore, the impact of salt stress on the sugar export capacity of source leaves was assessed by determining the sugar exudation rate of fully expanded source leaves (Fig. 5). Sucrose exudation rate was reduced by 27% in StSXD1:RNAi-21 and by 36% in StSXD1:RNAi-22 leaves compared to wild-type plants in control conditions. The sucrose exudation rate was not significantly altered in wild-type plants under salt stress, while tocopherol-deficient plants exhibited a >80% decrease in export rate. Thus, sugar export rate was strongly diminished in salt-stressed SXD:RNAi leaves, despite diminished callose deposition in the vasculature.

As a consequence of decreased sugar export in SXD1:RNAi source leaves under salt stress, a 60% decline in tuber weight of the transgenic plants compared to the wild type was observed early during tuberization at 20 days post-stress initiation (dpi) (Table 1). In control conditions, the tuber biomass of the transgenic plants was diminished by only 48% compared to the wild type at the same time point. The decrease in tuber yield of the transgenic plants became more pronounced at the end of the life cycle. SXD:RNAi plants exhibited a yield penalty of ~79–83% under stress vs 42% in control conditions at final harvest (Table 1). Tuber starch content remained comparable between all genotypes within the same treatment, both at 20 and 58 days after the onset of stress (Supplementary Figure S1). Nevertheless, tuber starch content decreased 2-fold in stress compared to control conditions in all genotypes. In contrast, aerial biomass, as measured 20 days after the onset of treatment, was similar in the three genotypes in control and salt-stress conditions (it was even higher in StSXD1:RNAi-21 than in wild-type controls). As a consequence, the shift in favour of aerial versus tuber biomass was more pronounced in the transgenic plants under salt-stress conditions (Table 1).

**Table 1.** Aerial and tuber biomass of tocopherol-deficient potato plants at two time points during salt-stress exposure^a^

	Treatment		
	Genotype	Control	Salt stress
Aerial biomass (g) 20 dpi	Wild type	52.0±2.0a	53.2±4.5a
	SXD1:RNAi 21	58.7±3.8b	56.6±7.7a
	SXD1:RNAi 22	51.5±4.3a	53.8±5.5a
Tuber biomass (g) 20 dpi	Wild type	67.0±3.2a	39.3±2.6a*
	SXD1:RNAi 21	41.5±1.7b	15.8±3.6b*
	SXD1:RNAi 22	28.2±3.6c	15.6±2.2b*
Tuber biomass (g) 58 dpi	Wild type	132.8±6.4a	16.3±1.3a*
	SXD1:RNAi 21	77.7±8.8b	2.8±2.8b*
	SXD1:RNAi 22	76.8±8.6b	3.5±2.0b*
Shoot / Tuber ratio 20 dpi	Wild type	0.78±0.01a	1.36±0.16a*
	SXD1:RNAi 21	1.42±0.10b	3.46±0.06b*
	SXD1:RNAi 22	1.84±0.18c	3.54±0.80b*

^a^ Aerial biomass was measured one day after leaf sampling (20 dpi) and tuber biomass was measured at 20 dpi and at the end of the experiment (58 dpi). Data represent the means ± SD of 4–5 individual plants. Data were analysed by *t*-test: significant differences among genotypes within a treatment are indicated by different letters for each parameter, and significant differences between control and salt stress within a genotype are indicated by an asterisk (*P* < 0.05).

### Tocopherol-deficient potato leaves exhibit less sodium accumulation, but a higher soluble antioxidant capacity during salt challenge

**Fig. 6.**
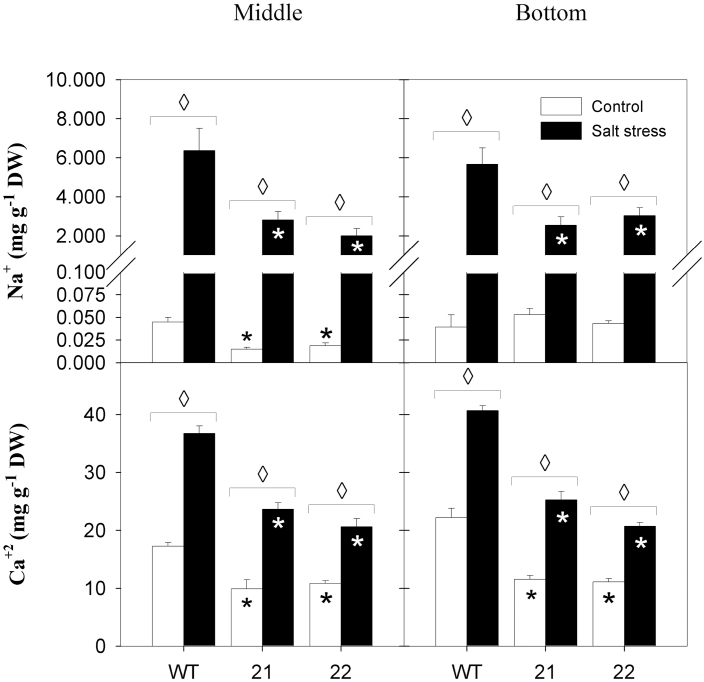
Effects of salt treatment on sodium and calcium content in source leaves. Sodium (top panels) and calcium contents (bottom panels) are depicted on a dry weight (DW) basis. Left panels represent middle leaves (leaf 8); right panels represent bottom leaves (leaf 11). Samples were collected 19 days after the onset of treatments (salt stress, black bars; control, white bars) and data represent the mean ± SE of four individual plants. Data were analysed by *t*-test; significant differences between the transgenic lines and the wild type (WT) within a treatment are indicated by a black asterisk (control treatment) or a white asterisk (stress treatment), while diamonds indicate significant differences between control and salt stress within a genotype (*P* < 0.05).

Since the negative impact of tocopherol deficiency on sugar export and tuber yield was exacerbated under salt treatment, we assessed whether the physiology of tocopherol-deficient leaves was more sensitive to salt challenge and whether an elevated degree of oxidative stress might explain the altered sugar response of transgenic leaves. The CO_2_ assimilation rate of SXD1:RNAi source leaves was significantly reduced by 40–60% in the transgenic plants under salt-stress conditions (Table 2). The transpiration rate was diminished in salt-stressed transgenic plants in comparison to stressed wild-type plants (Table 2), and concomitantly the contents of xylem-mobile Na^+^ and Ca^2+^ were 2- to 3-fold reduced in SXD1:RNAi source leaves (Fig. 6). Consequently, the K^+^/Na^+^ ratio was higher in both middle and bottom leaves of salt-stressed transgenic plants, indicating that the foliar ionic balance was less disturbed in SXD1:RNAi leaves compared to the wild type (Supplementary Figure S2). However, leaf osmolality in middle and bottom source leaves did not exhibit significant changes across genotypes and treatments (Supplementary Figure S3).

**Table 2.** Gas-exchange and PSII efficiency parameters of *StSXD1*-silenced potato plants after 14-days’ exposure to salt stress^a^

	Treatment		
	Genotype	Control	Salt stress
A (μmol CO_2_ m^–2^ s^–1^)	Wild type	5.70±1.80a	2.40±0.59a*
	SXD1:RNAi 21	4.43±2.28a	0.85±0.13b*
	SXD1:RNAi 22	4.95±1.62a	1.38±0.64b*
E (mmol m^–2^ s^–1^)	Wild type	1.36±0.68a	0.43±0.12a*
	SXD1:RNAi 21	1.12±0.91a	0.20±0.05b*
	SXD1:RNAi 22	1.05±0.43a	0.27±0.11b*
ETR	Wild type	69.1±2.3a	59.3±5.5a*
	SXD1:RNAi 21	71.2±6.4a	51.0±6.4a*
	SXD1:RNAi 22	71.5±7.2a	56.5±8.5a*
Fv/Fm	Wild type	0.797±0.016a	0.789±0.017a
	SXD1:RNAi 21	0.784±0.011a	0.759±0.075a
	SXD1:RNAi 22	0.806±0.024a	0.794±0.026a

^**a**^ Data correspond to middle leaves (leaf 8) and represent the means ± SD of 4–5 individual plants. Data were analysed by *t*-test: significant differences among genotypes within a treatment are indicated by different letters for each parameter, and significant differences between control and salt stress within a genotype are indicated by an asterisk (*P* < 0.05).

**Fig. 7.**
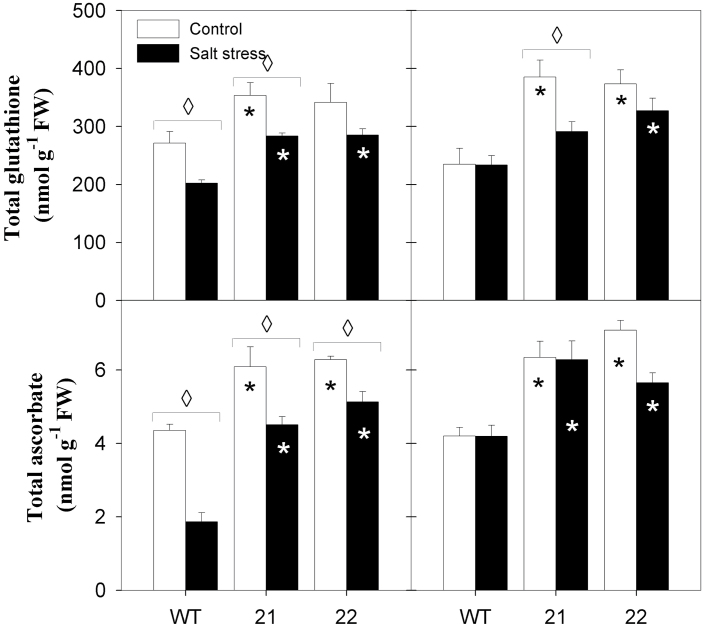
Effects of salt treatment on the pool size of foliar soluble antioxidants in source leaves of *StSXD1*-silenced potato plants. Total glutathione (top panels) and total ascorbate content (bottom panels) are depicted. Left panels represent middle leaves (leaf 8); right panels represent bottom leaves (leaf 11). Samples were collected 19 days after the onset of treatments (salt stress, black bars; control, white bars) and data represent the mean ± SE of four individual plants. Data were analysed by *t*-test; significant differences between the transgenic lines and the wild type (WT) within a treatment are indicated by a black asterisk (control treatment) or a white asterisk (stress treatment), while diamonds indicate significant differences between control and salt stress within a genotype (*P* < 0.05).

The pool sizes of the major water-soluble antioxidants, ascorbate and glutathione, were elevated in middle and bottom source leaves of the transgenic plants compared to the wild type, in control conditions already (Fig. 7). Salt stress caused a decrease in total glutathione and, to a greater extent, total ascorbate content of middle wild-type leaves, while total glutathione and ascorbate contents were comparable in stressed and non-stressed bottom wild-type leaves. Ascorbate and glutathione pool sizes were also diminished in tocopherol-deficient plants upon salt treatment, but commonly both pools remained larger compared to wild-type controls (Fig. 7). The redox state of the ascorbate and glutathione pools were very similar across genotypes and treatments, with glutathione showing values higher than 90%, while ascorbate redox state ranged around 80% (Supplementary Figure S4). MDA levels were not significantly different between the genotypes irrespective of treatment and, in addition, MDA levels were not increased upon salt stress in the transgenic plants, indicating the absence of excessive lipid peroxidation in salt-stress conditions (Supplementary Figure S4).

Furthermore, transgenic plants survived longer than wild-type plants under salt stress, as shown in Supplementary Figure S5, which represents plants 42 days after the onset of treatment. At that time, stressed wild-type plants were dead, while all tocopherol-deficient plants, despite showing severe wilting symptoms, still exhibited a few pale green leaves (see arrows in Supplementary Figure S5).

In summary, SXD1:RNAi potato plants were more tolerant towards salt stress compared to the wild type, which can be accounted for by reduced Na^+^ accumulation in leaves and elevated soluble antioxidant pools. However, the transgenic plants exhibited a much more pronounced yield penalty when challenged with saline conditions compared to the wild type.

### Central carbon and amino acid metabolism and sucrose-dependent gene expression are altered in salt-stressed SXD1:RNAi

**Fig. 8.**
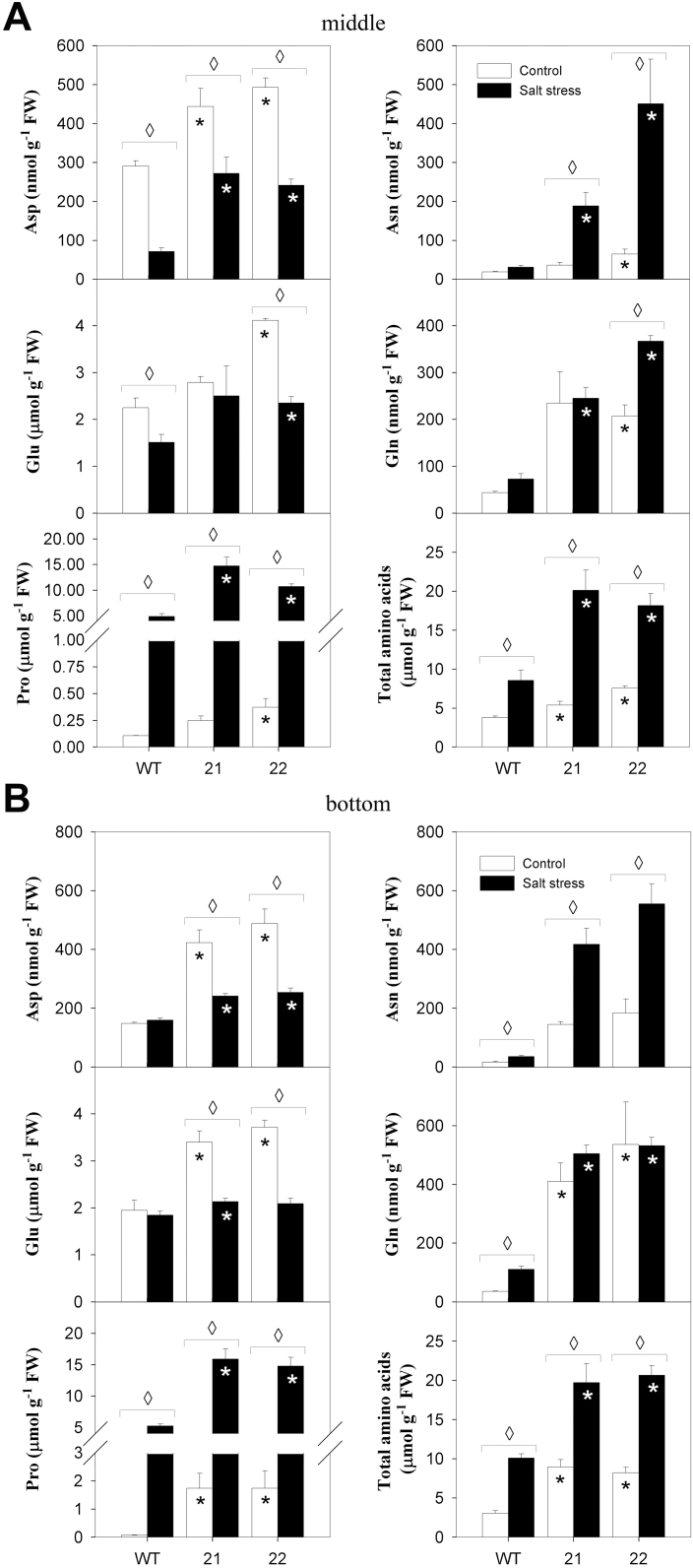
Content of free amino acids in source leaves of tocopherol-deficient plants. (A) Amino acid contents in middle leaves (leaf 8). (B) Amino acid contents in bottom leaves (leaf 11). Total amino acid levels represent the sum of single free amino acid contents determined by HPLC. Samples were collected 19 days after the onset of treatments (salt stress, black bars; control, white bars) and data represent the mean ± SE of four individual plants. Data were analysed by *t*-test; significant differences between the transgenic lines and the wild type within a treatment are indicated by a black asterisk (control treatment) or a white asterisk (stress treatment), while diamonds indicate significant differences between control and salt stress within a genotype (*P* < 0.05).

Despite the observed lower Na^+^ uptake rates and increased antioxidant capacity, increased accumulation of compatible solutes could also account for the elevated tolerance of SXD:RNAi plants towards salt stress. We had already found that the contents of osmotically active soluble sugars were increased by about 40% in stressed SXD:RNAi leaves compared to the wild type (Fig. 1). Furthermore, Pro accumulation in stressed transgenic plants was 2- to 3-fold higher compared to stressed wild-type plants (Fig. 8). Pro accounted for up to 70% of the total foliar free amino acid pool in SXD:RNAi leaves, which corresponds to a 1.5- to 2- fold increase of Pro relative to the wild type.

Likewise, the contents of the major free amino acids Gln, Asn, and Asp were elevated in SXD1:RNAi leaves compared to the wild type in both control and stress conditions (Fig. 8). While the accumulation of these major amino acids was more significant in salt-stressed transgenic plants, the contents of most TCA cycle intermediates dropped more pronouncedly compared to wild-type leaves (Supplementary Figure S6), which may reflect elevated carbon flux from carboxylate precursors into the amino acid pool. Asparagine contents were increased most pronouncedly in SXD:RNAi leaves in comparison to the wild type (Fig. 8): Asn content was elevated 2–3 times in SXD:RNAi source leaves in control conditions and even 6–15 times higher in salt-stressed transgenic plants.

**Fig. 9.**
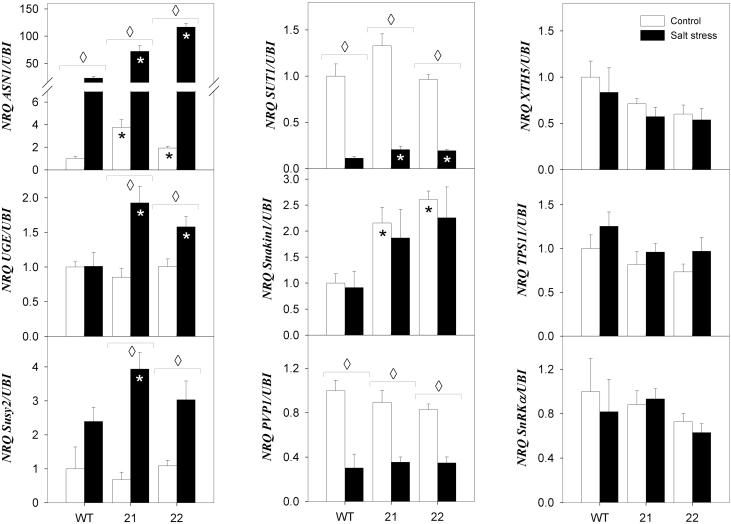
Relative transcript accumulation of genes involved in sucrose export and SnRK1 target genes in source leaves of *StSXD1*-silenced potato plants. The transcript levels of the genes indicated were measured in middle leaves (leaf 8) of tocopherol-deficient and wild-type (WT) potato plants after 19 days of treatment (salt stress, black bars; control, white bars) with ubiquitin as the reference gene. Data represent the mean ± SE of 4–5 individual plants. Left column (responding SnRK1 target genes): *ASN1* (asparagine synthetase), *UDPglcE* (UDP-glucose epimerase), and *SuSy2* (sucrose synthase). Middle column (genes involved in sucrose export): *SUT1* (H^+^-sucrose transporter), *Snakin1* (*SN1*), and *PVP1* (vacuolar H^+^-pyrophosphatase). right column (non-responding SnRK1 target genes): *XTH5* (xyloglucan endotransglucosylase-hydrolase), *TPS11* (trehalose 6-phosphate synthase), and *SnRKα* (α subunit of SnRK1, sugar non-fermenting related kinase1). Data were analysed by *t*-test; significant differences between the transgenic lines and the wild type within a treatment are indicated by a black asterisk (control treatment) or a white asterisk (stress treatment), while diamonds indicate significant differences between control and salt stress within a genotype (*P* < 0.05).

As the asparagine synthase gene *ASN1* is known to represent a SnRK1 target gene, the altered effect of salt stress on free Asn content in SXD1:RNAi leaves prompted us to analyse SnRK1-mediated regulation of metabolism. In Fig. 9, the transcript accumulation of six SnRK1 target genes is shown: *ASN1*, *UGE*, *SuSy2*, *XTH5*, *TPS11*, and *SnRKα* (coding for sugar-regulated asparagine synthetase, UDP-glucose epimerase, sucrose synthase, xyloglucan endotransglucosylase-hydrolase, and trehalose 6-phosphate synthase isoforms, respectively (see Debast *et al.*, 2011). The *Arabidopsis* homologues of *ASN1*, *SuSy2*, *SnRKα*, and *XTH5* were found to be regulated via the transcription factor bZIP11 (Hanson *et al.*, 2008). A strong upregulation of *ASN1* and *SuSy2* transcript amounts was observed in all genotypes upon salt stress, and *ASN1* and *SuSy2* transcript levels were induced much more strongly in stressed SXD1:RNAi than in wild-type plants. (Fig. 9). *UGE* was induced in tocopherol-deficient plants upon salt exposure, but not in wild-type plants. In contrast, *XTH5*, *TPS11*, and *SnRKα* transcripts did not show significant differences across treatments or genotypes (Fig. 9). Furthermore, the content of trehalose-6-phosphate, which is thought to correlate with cellular sucrose availability (Lunn *et al.*, 2006), was elevated about 2-fold in SXD1:RNAi compared to wild-type source leaves in all conditions (Supplementary Figure S6). Taken together, our data indicate a discrepancy between individual indicators of cellular sucrose availability, i.e. SnRK target gene expression and trehalose-6-phosphate content.

We therefore assessed whether the regulation of sucrose export from source leaves is also altered in SXD1:RNAi. The transcript level of the single copy H^+^/sucrose cotransporter *SUT1*, which controls apoplastic phloem loading (Riesmeier *et al.*, 1993), was strongly diminished upon salt stress, but there were no consistent differences between tocopherol replete and depleted genotypes (Fig. 9). In contrast, the *SUT1-*interacting protein *Snakin-1* (Kruegel *et al*., 2012) was induced at the transcript level in SXD:RNAi leaves under all conditions (Fig. 9). Cell wall-bound and soluble invertase activity, which can also efficiently counteract phloem loading (Rolland *et al.*, 2006), were not significantly altered between wild-type and transgenic plants in control and salt-stress conditions (Supplementary Figure S7). Similarly, transcript amounts of the vacuolar H^+^-pyrophosphatase, which might prevent sucrose utilization and phloem loading (Lerchl *et al.*, 1995), were diminished to a similar extent in all genotypes under salt stress (Fig. 9). Our data leave *Snakin-1* as the only potential candidate that may affect the rate of sucrose export from SXD:RNAi source leaves upon salt stress.

### ACC, SA, and cytokinin levels are altered in source leaves of tocopherol-deficient potato

**Fig. 10.**
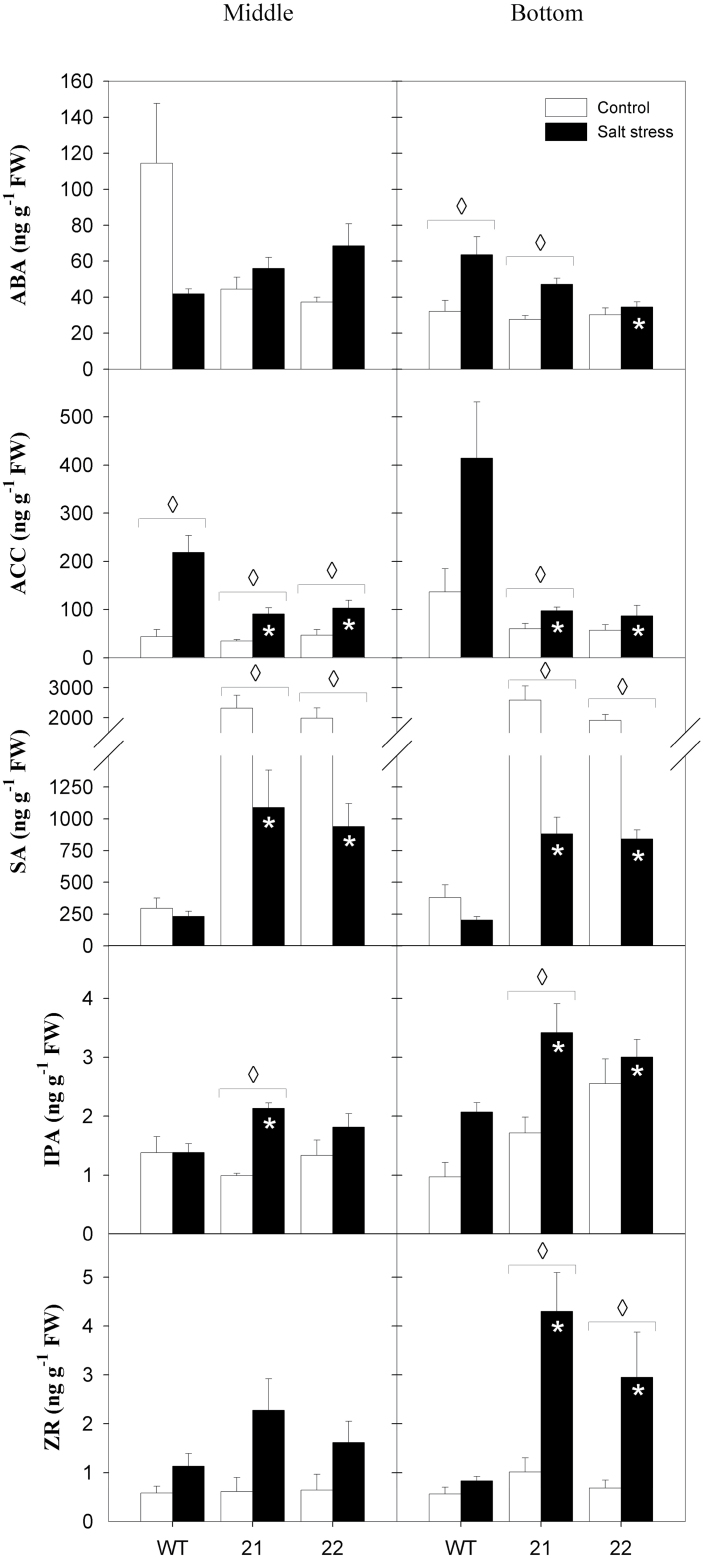
Phytohormone contents in source leaves of tocopherol-deficient potato plants exposed to 19 days’ salt treatment. Leaf content of ABA, ACC, SA, and of the cytokinins IPA and ZR are shown from top to bottom. Left panels represent middle leaves (leaf 8); right panels represent bottom leaves (leaf 11). Black bars, salt-stress treatment; white bars, control treatment. Data represent the mean ± SE of four individual plants. Data were analysed by *t*-test; significant differences between the transgenic lines and the wild type within a treatment are indicated by a black asterisk (control treatment) or a white asterisk (stress treatment), while diamonds indicate significant differences between control and salt stress within a genotype (*P* < 0.05).

In order to gain more insight into the altered physiology of tocopherol-deficient plants, the levels of a range of hormones related to stress responses as well as to plant growth and development were measured. ABA accumulation was elevated upon salt stress only in bottom leaves, which was more pronounced in the wild type compared to SXD:RNAi leaves (Fig. 10). While the ethylene precursor ACC displayed a 5- and 3-fold increase in middle and bottom leaves of wild-type plants exposed to salt stress, respectively, transgenic plants showed a less dramatic increase in ACC content, reaching 40% (middle leaves) and 20% (bottom leaves) of wild-type ACC levels under salt stress (Fig. 10). In parallel, SXD1:RNAi lines contained 5- to 8-fold more SA than wild-type plants in control conditions. Salt stress did not cause a substantial decrease in SA levels in wild-type leaves, while a 40–65% decline of SA content was found in stressed middle and bottom leaves of transgenic plants compared to control conditions. As a result, the difference in SA levels between wild-type and transgenic plants was lowered upon salt treatment, although they remained significantly higher in the transgenic plants. JA levels were low compared to the above-mentioned stress hormones, and differences were not found between the three genotypes (Supplementary Figure S8). Levels of the cytokinins IPA and ZR were higher in bottom leaves of stressed transgenic plants compared to wild-type plants (Fig. 10). Diminished ACC and SA as well as elevated cytokinin contents all point towards delayed senescence in salt-stressed SXD1:RNAi leaves, which might simply be caused by a diminished hexose/sucrose ratio in stressed transgenic plants (also observed in Lara *et al.*, 2004; Kocal *et al.*, 2008).

## Discussion

### SXD:RNAi potato lines as a tool to study the potential causes of a sugar export block

In previous work by Hofius *et al.* (2004), it was observed that tocopherol-deficient SXD:RNAi potato plants exhibited a photoassimilate export-defective phenotype that coincided with callose deposition in the phloem of source leaves, similar to what had been observed for the maize tocopherol deficient *sxd1* mutant previously (Botha *et al.*, 2000; Provencher *et al.*, 2001). On the other hand, tocopherol-deficient tobacco and *Arabidopsis* plants did not exhibit such a sugar export block in standard growth conditions at regular PFD (Maeda *et al.*, 2006; Abbasi *et al.*, 2009). This difference between species can be explained by the fact that potato and maize exhibit a high rate of sugar export from source leaves due to the presence of strong systemic carbon sinks, while *Arabidopsis* and tobacco source leaves have lower sugar export rates per leaf area (see also Abbasi *et al.*, 2009).

When growing the same tocopherol-deficient SXD:RNAi potato lines at a photon flux density (PFD) of 250 μmol m^–2^ s^–1^ (this study) instead of 500 μmol m^–2^ s^–1^ (as in Hofius *et al.*, 2004), starch accumulation in unstressed source leaves of the transgenic plants was absent, and only a 2.5-fold accumulation of soluble sugars and limited callose accumulation in the vasculature were observed compared to the wild type. This suggests that callose plugging in the vasculature of SXD:RNAi potato may depend on incident light intensity. We aimed at exploiting these permissive growth conditions to examine whether the disturbance of foliar physiological processes by salt stress can induce a sugar export block in tocopherol-deficient potato plants.

Tocopherol-deficient *Arabidopsis vte2* mutants showed a sugar export block during cold acclimation, which was dependent on fatty acid desaturation in the ER-derived phospholipid pool by FAD2 (fatty acid desaturase2) (Maeda *et al.*, 2008). As this stress-inducible sugar export block in *vte2* was independent of light intensity, the degree of photoinhibition, or lipid peroxidation, it may thus be linked to non-antioxidant functions of tocopherols (Maeda *et al.*, 2008). Since increased synthesis of PUFAs is important for acclimation to both cold and salt stress in plants (e.g. Maeda *et al.*, 2008; Zhang *et al.*, 2012), salt challenge of SXD:RNAi potato plants was thought to provide information as to whether non-oxidant functions of tocopherol are important for salt-stress acclimation in potato leaves.

### The reduction in sugar export rate in tocopherol-deficient potato leaves is not caused by excessive callose plugging

The content of soluble sugars was higher in SXD:RNAi source leaves compared to wild-type plants in salt stress and, in addition, starch mobilization in transgenic plants was abolished under salt-stress conditions. However, soluble sugars and starch contents of stressed transgenic plants did not exceed the level found in control conditions. Therefore, the response of major leaf carbohydrates in SXD transgenic plants to salt stress does not compare to what is described as a sugar export block in previous reports (Botha *et al.*, 2000; Hofius *et al.*, 2004; Maeda *et al.*, 2006). In salt stress, there is neither an excessive accumulation of leaf carbohydrates, nor pronounced plugging of plasmodesmata in the leaf vasculature of the transgenic plants. Even if tocopherol-deficiency had caused limited phospholipid desaturation in salt-stressed SXD:RNAi leaves as described for cold-stressed *Arabidopsis vte2* mutants (Maeda *et al.*, 2008), this did not lead to a sugar export block phenotype. Therefore, sugar export in the potato transgenic plants must be impaired by a different mechanism.

Sugar export in tocopherol-deficient source leaves was diminished by 80% upon salt stress and nocturnal sugar and starch levels were higher than in stressed wild-type plants, indicating an elevated retention of carbohydrates in SXD:RNAi source leaves. Furthermore, the substantial sugar export deficiency of salt-stressed transgenic plants was evident through a massively diminished exudation rate and caused a substantial tuber yield penalty. Thus, we tried to explain this phenomenon at the molecular level. First, we assessed whether reduced apoplastic phloem loading could explain the marked decline in sugar export in salt-stressed, tocopherol-deficient plants by analysing the transcript abundance of the sugar transporter *SUT1* that is responsible for phloem loading in potato (Riesmeier *et al.*, 1993) and represents a single-copy gene. *SUT1* was strongly downregulated upon salt stress in all three genotypes, although *SUT1* transcript levels remained significantly higher in the transgenic plants compared to the wild type. Once an increased *SUT1* transcript abundance is unlikely to result in less apoplastic phloem loading in salt-challenged SXD:RNAi leaves, we looked at the *SUT1-*interacting proteins *PDI* and *Snakin1* (Krügel *et al.*, 2012), which are thought to have the potential to modulate SUT1 activity by integrating redox and/or defence signals (Krügel and Kühn, 2013). However, direct experimental evidence in support of these assumed roles is lacking. *Snakin1* (*SN1*) transcript amounts were increased 2-fold in SXD:RNAi leaves under all conditions, while PDI transcript amounts remained unaltered across genotypes and conditions (not shown). Strong overexpression of the small cysteine-rich SN1 protein in potato had only minor effects on morphology and primary metabolism in the absence of abiotic stress (Nahirnak *et al*., 2012). Notably, proline contents were found to be increased in *SN1* overexpressors compared to controls (Nahirnak *et al*., 2012), which might indicate increased carbon allocation into this osmoprotectant that may occur at the expense of sucrose export. It seems valuable to test this assumption by studying salt- and drought-stress tolerance of *SN1-*overexpressing potato plants.

We also investigated other key functions that can influence sucrose export. Both cell wall invertase (cw-INV) and soluble invertase can counteract apoplastic loading by cleaving sucrose in the apoplast or by stimulating sucrose import and cleavage in the vacuole, respectively (Rolland *et al.*, 2006). Both activities seem to be diminished upon salt stress, but consistent differences were absent between the genotypes.

An induction of vacuolar H^+^-pyrophosphatase is observed in the halophyte *Thelungiella halophila* in saline conditions (Sun *et al.*, 2010). Although the *Arabidopsis* homologue AVP1 is not induced upon salt stress, overexpression of AVP1 homologues from wheat, *Arabidopsis* and *T. halophila* were able to increase salt-stress tolerance in plants (e.g. Gaxiola *et al.*, 2001; Gao *et al.*, 2006). Elevated tonoplast H^+^-pyrophosphatase is thought to increase the tonoplast membrane potential to drive the import of potassium into the vacuole by proton-coupled uptake systems (Blumwald *et al.*, 2000). It is reasonable to assume that the cytosol might be deprived of pyrophosphate by elevated H^+^-pyrophosphatase activity, thereby hampering phloem loading of sucrose in companion cells that rely on low cytosolic pyrophosphatase activity to efficiently utilize sucrose (Lerchl *et al.*, 1995). However, the potato homologue of AVP1 was transcriptionally repressed by salt stress to a similar extent in all three genotypes, disproving this attractive hypothesis.

Once we could not identify substantial differences in these major players of sucrose export, we checked whether an imbalance in cellular sugar signalling might account for reduced sucrose export in salt-stressed SXD:RNAi source leaves.

### Sugar signalling might attenuate sucrose export in tocopherol-deficient source leaves

In plants, SnRK1s are highly conserved protein kinases closely related to SNF1 in yeast and AMPK in animals (Halford *et al.*, 2003), which play a key role in the regulation of carbon metabolism and energy balance. Upon energy depletion caused by abiotic stress situations, darkness, or nutrient deprivation, SnRK1s trigger extensive transcriptional activation of major catabolic pathways to provide alternative sources of energy and metabolites (Baena-González *et al*, 2007). To assess whether SnRK1 signalling is altered in SXD:RNAi leaves, we determined the transcript levels of several genes that were shown to be potential SnRK1 targets in potato tubers (Debast *et al.*, 2011). Three of the eight target genes, *AsnS1*, *SuSy2*, and *UDPglcE*, showed higher transcript levels in tocopherol-deficient plants than in wild-type plants under salt stress. Despite a very substantial induction of *ASN1* upon salt stress, transcripts of all three genes were <2-fold elevated in SXD:RNAi leaves compared to the wild type under stress conditions. Three other SnRK1 targets, *XTH5*, *TPS11*, and *SnRKα* (Fig. 9), as well as *SnRKγ* and *SEN1* (not shown), were not differentially expressed across genotypes or conditions. This either indicates that SnRK1 signalling does not play a role in salt-stressed potato leaves, or that SnRK1 targets may differ between leaf and tubers (see Debast *et al.*, 2011). In support of a role of SnRK1 signalling during salt stress, it has been shown that SnRK1 is engaged in the regulation of vacuolar potassium transport during salt challenge in the ice plant *Mesembryanthemum crystallinum* (Chiang *et al.*, 2013), that the SnRK1 target transcription factor bZIP11 is induced in salt-stressed *Arabidopsis* leaves (Weltmeier *et al.*, 2009), and that SnRK1 is activated in other abiotic stress conditions like drought, cold, or hypoxia (see O’Hara *et al.*, 2013 for a compilation), which also influence the cellular osmotic potential. While the accumulation of sugars and the signalling metabolite T6P declined in SXD:RNAi leaves upon salt stress, *AsnS1*, *SuSy2*, and *UDPglcE* were increased about 2-fold more in the transgenic plants than in the wild type. Thus, the response of these three SnRK1 target genes is consistent with a decline in sugar and T6P upon salt stress in the transgenic plants.

Based on our results, we propose a chain of events that can explain the strong reduction in sucrose export in SXD:RNAi leaves during salt stress. The extensive and continuous accumulation of sugars in the vacuole of salt-stressed leaf cells probably leads to a drop in cytosolic sucrose concentration, which in turn engages SnRK1 signalling to relay carbon shortage. Tocopherol-deficient leaves apparently produce a stronger SnRK1 signal than the wild type, which might result in a more pronounced attenuation of sucrose export from SXD:RNAi source leaves. While the transcriptional repression of SUT1 as a key player of phloem loading is similar in all salt-stressed genotypes, SUT1 activity might be diminished in the transgenic plants due to interaction with other proteins like Snakin-1. This hypothesis seems especially attractive, since the cysteine-rich protein Snakin-1 has the potential to integrate redox signals (Krügel and Kühn, 2013), which might already be altered in unstressed SXD:RNAi leaves due to increased pools of the soluble antioxidants ascorbate and glutathione. Increased retention of sugars in salt-stressed SXD:RNAi leaf cells does not ameliorate sugar availability, but rather dampens photosynthetic carbon assimilation. In addition, nocturnal starch mobilization may also be abrogated in the transgenic plants as a consequence of altered SnRK1 signalling (as summarized in O’Hara *et al.*, 2013).

It remains a challenge for future studies to resolve how tocopherol deficiency effects increased sensitivity of SXD:RNAi plants to SnRK1 signalling during salt-stress adaptation.

### SXD:RNAi leaves remain more virescent under salt stress

In our study, major differences between tocopherol-deficient and wild-type potato plants were displayed under salt-stress conditions. Because tocopherol is a well-known antioxidant, with a major role in protecting unsaturated fatty acids and the photosynthetic apparatus from oxidative damage, it would be expected that leaves of tocopherol-deficient plants suffer from enhanced oxidative stress upon salt challenge. However, salt-stressed SXD:RNAi leaves showed unaltered photochemical efficiency of PSII and MDA levels as well as comparable ascorbate and glutathione redox states relative to wild-type plants. However, tocopherol deficiency in SXD1:RNAi plants was compensated by an increase in ascorbate and glutathione pools during salt stress. A similar compensatory response was found by Kanwischer *et al.* (2005) in *Arabidopsis vte1* mutants. Taken together, this all indicates that leaves of tocopherol-deficient plants did not suffer from more severe oxidative stress than wild-type plants under saline conditions, as was also evident from the phenotype of the stressed plants. An analysis of phytohormone contents supports the macroscopic observation that leaf senescence is delayed in salt-stressed transgenic plants compared to the wild type. It is well known that SA can accumulate in damaged or necrotic tissue during leaf senescence, but SA contents were reduced by 50% in salt-stressed leaves of SXD1:RNAi in comparison to unstressed controls. Furthermore, the contents of ACC and ABA, which can both act as triggers of leaf senescence (Gan and Amasino, 1997), remained low in salt-stressed SXD:RNAi leaves, while their contents were particularly elevated in bottom leaves of salt-stressed, wild-type plants. Concomitantly, the cytokinin zeatin riboside was most pronouncedly elevated in bottom leaves of tocopherol-deficient plants under salt challenge. The increased longevity of SXD:RNAi leaves under salt stress can be explained by a synergism of increased soluble antioxidants, reduced sodium uptake as a consequence of a diminished transpiration rate, and elevated proline contents.

## Conclusion

Our study has led to two major results. First, it has revealed that sugar export from tocopherol-deficient leaves can be reduced in the absence of excessive callose plugging of plasmodesmata in the vasculature. Our data favour the idea that an increased sensitivity to SnRK1 signalling impedes sucrose export in SXD:RNAi leaves. Second, despite a severe reduction in tuber yield, tocopherol-deficient potato leaves did not exhibit physiological signs of elevated oxidative stress upon salt challenge. The delayed senescence of the salt-stressed transgenic plants can be explained by reduced sodium uptake, and by enhanced accumulation of the osmoprotectant proline and soluble antioxidants.

It will remain a challenge for the future to entirely unravel the molecular basis of reduced sucrose export in these salt-stressed, tocopherol-deficient potato plants.

## Supplementary material

Supplementary Figure S1. Tuber starch content of tocopherol-deficient potato plants at two time points during salt-stress exposure.

Supplementary Figure S2. Effects of salt treatment on potassium content and the potassium/ sodium ratio in source leaves.

Supplementary Figure S3. Leaf osmolality of salt-treated and control plants.

Supplementary Figure S4. Effects of salt treatment on the redox state of foliar soluble antioxidants and lipid peroxidation in source leaves of *StSXD1*-silenced potato plants.

Supplementary Figure S5. Phenotype of 11-week-old tocopherol-deficient potato plants compared to the wild type after the end of the salt-stress treatment.

Supplementary Figure S6. Contents of intermediates of central carbon metabolism in source leaves of SXD1:RNAi transgenic potato plants upon salt exposure.

Supplementary Figure S7. Soluble and cell-wall bound invertase activity in *StSXD1*-silenced potato plants after 19 days of salt stress.

Supplementary Figure S8. Phytohormone contents in source leaves of tocopherol-deficient potato plants exposed to 19 days salt treatment.

## Funding

Part of this work was funded by a travel grant to María Amparo Asensi-Fabado from the Deutsche Forschungsgemeinschaft (DFG) in the framework of the SFB796.

## Supplementary Material

Supplementary Data
