# Supplementary material for: Tocopherol deficiency reduces sucrose export from salt-stressed potato leaves independently of oxidative stress and symplastic obstruction by callose
Source: J Exp Bot. 2014 Nov 26;66(3):957–71. doi: 10.1093/jxb/eru453 (PMC4321552; doi:10.1093/jxb/eru453)
Supplement: Supplementary Data [file supp_66_3_957__index.html]

Tocopherol deficiency reduces sucrose export from salt-stressed potato leaves independently of oxidative stress and symplastic obstruction by callose — Tocopherol deficiency reduces sucrose export from salt-stressed potato leaves independently of oxidative stress and symplastic obstruction by callose — Supplementary Data 

# Tocopherol deficiency reduces sucrose export from salt-stressed potato leaves independently of oxidative stress and symplastic obstruction by callose

## Supplementary Data

Data files

**Files in this Data Supplement:**

- Supplementary Data - Supplementary Data
