# Supplementary material for: Tocopherol deficiency reduces sucrose export from salt-stressed potato leaves independently of oxidative stress and symplastic obstruction by callose
Source: J Exp Bot. 2014 Nov 26;66(3):957–71. doi: 10.1093/jxb/eru453 (PMC4321552; doi:10.1093/jxb/eru453)
Supplement: Supplementary Data [file supp_eru453_jexbot133579_file001.pdf]

Supplementary Material

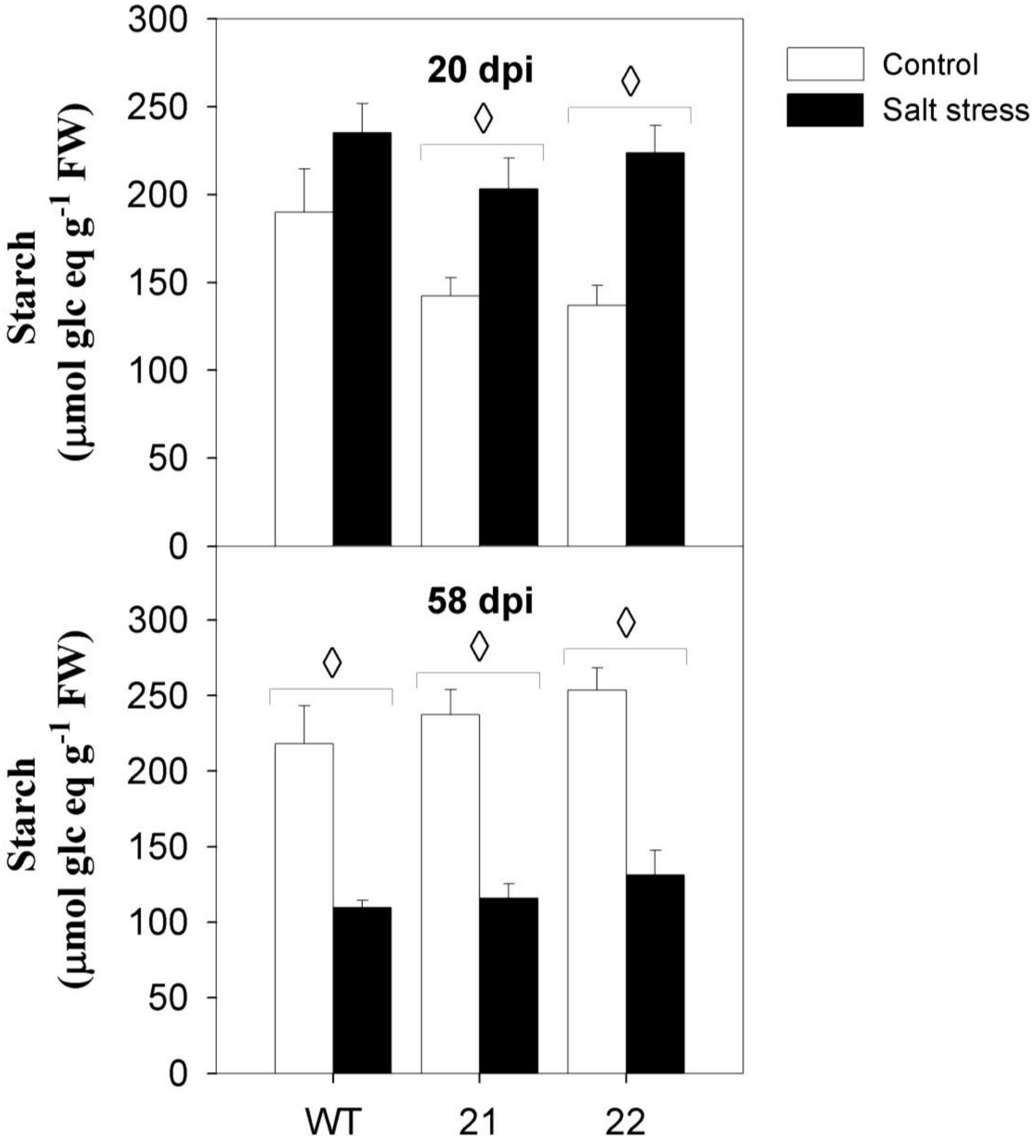

Figure S1. Tuber starch content of tocopherol-deficient potato plants at two time points during salt stress exposure. Measurements were performed with tubers of comparable size one day after leaf sampling (20 dpi, days post-stress initiation) and at the end of the experiment (58 dpi). Due to the strong reduction in tuber yield at the end of the experiment, only small tubers were investigated at both time points. Black bars, salt stress treatment; white bars, control treatment. Data represent the means ± SE of 4-5 individual plants. Statistically significant differences in starch content between salt stressed and control plants in a *t*-test with $P < 0.05$ are indicated by diamonds.

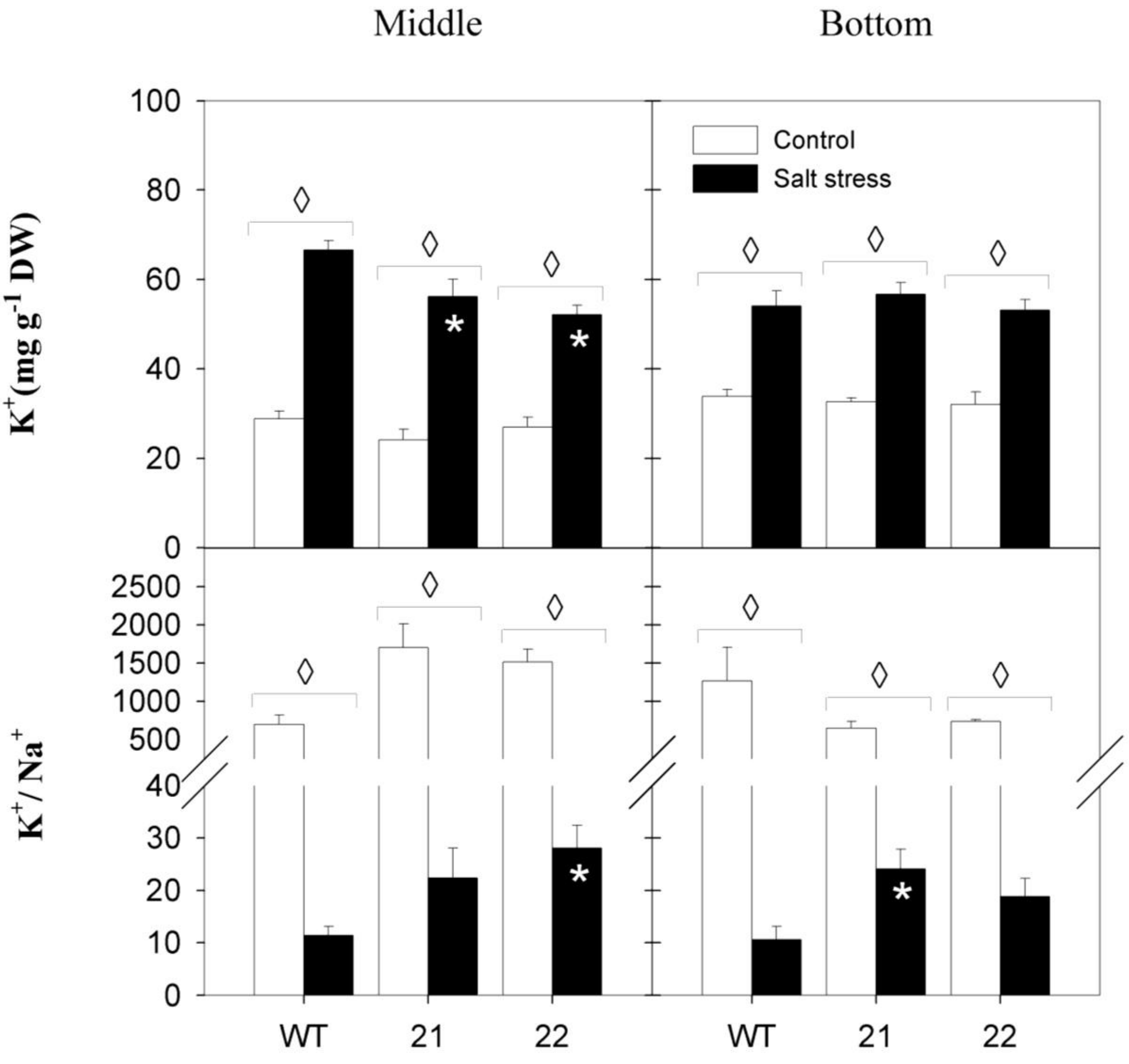

Figure S2. Effects of salt treatment on potassium content and the potassium/ sodium ratio in source leaves. Left panels represent middle leaves (leaf 8); right panels represent bottom leaves (leaf 11). Samples were collected 19 days after the onset of treatments (salt stress, black bars; control, white bars) and data represent the means ± SE of 4 individual plants. Data were analyzed by *t*-test; significant differences between the transgenic lines and the wild type within a treatment are indicated by a black asterisk (control treatment) or a white asterisk (stress treatment), while diamonds indicate significant differences between control and salt stress within a genotype ($P < 0.05$).

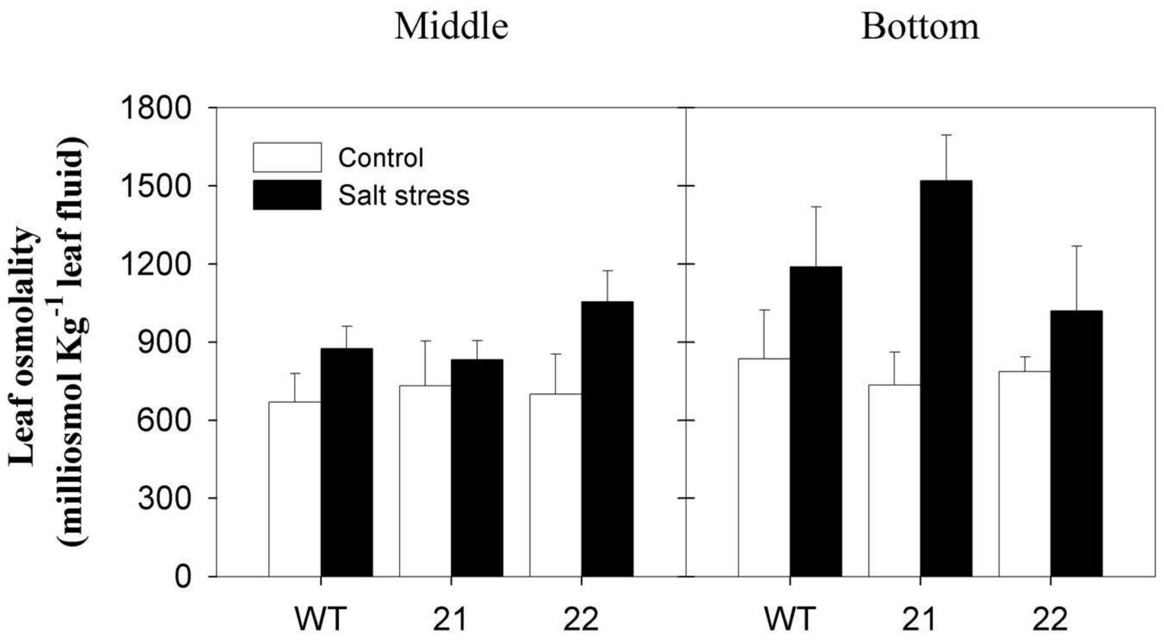

Figure S3. Leaf osmolality of salt treated and control plants. Left panels represent middle leaves (leaf 8); right panels represent bottom leaves (leaf 11). Samples were collected 19 days after the onset of treatments (salt stress, black bars; control, white bars) and data represent the means ± SE of 4 individual plants. No significant differences ($P < 0.05$) between the transgenics and the wild type or between treatments could be revealed by *t*-test.

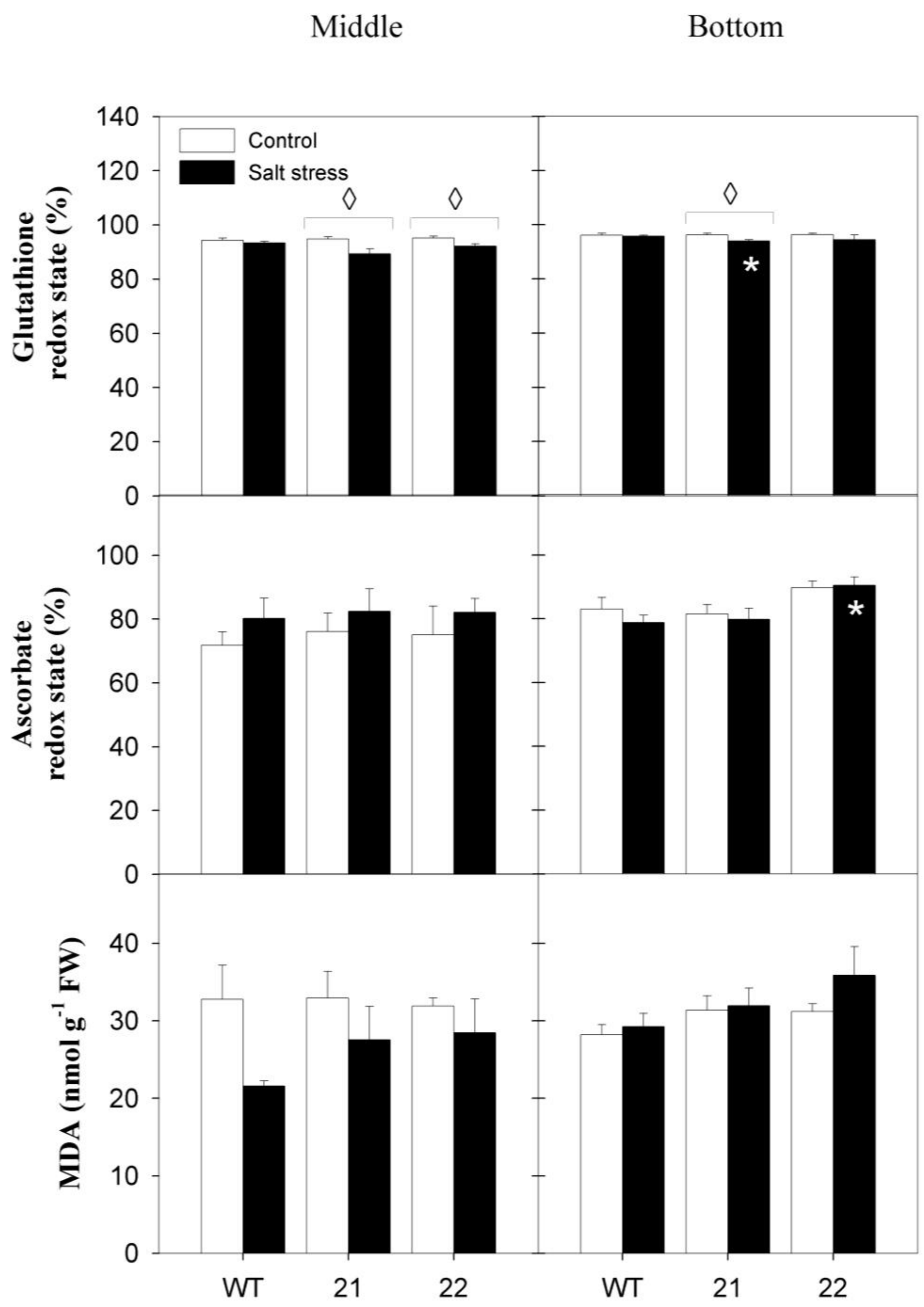

Figure S4. Effects of salt treatment on the redox state of foliar soluble antioxidants and lipid peroxidation in source leaves of *StSXD1*-silenced potato plants. Glutathione redox state (top panels), ascorbate redox state (middle panels) as well as malondialdehyde content as a measure of lipid peroxidation (bottom panels) are depicted. Left panels represent middle leaves (leaf 8); right panels represent bottom leaves (leaf 11). Samples were collected 19 days after the onset of treatments (salt stress, black bars; control, white bars) and data represent the means ± SE of 4 individual plants. Data were analyzed by *t*-test; significant differences between the transgenic lines and the wild type within a treatment are indicated by a black asterisk (control treatment) or a white asterisk (stress treatment), while diamonds indicate significant differences between control and salt stress within a genotype ($P < 0.05$).

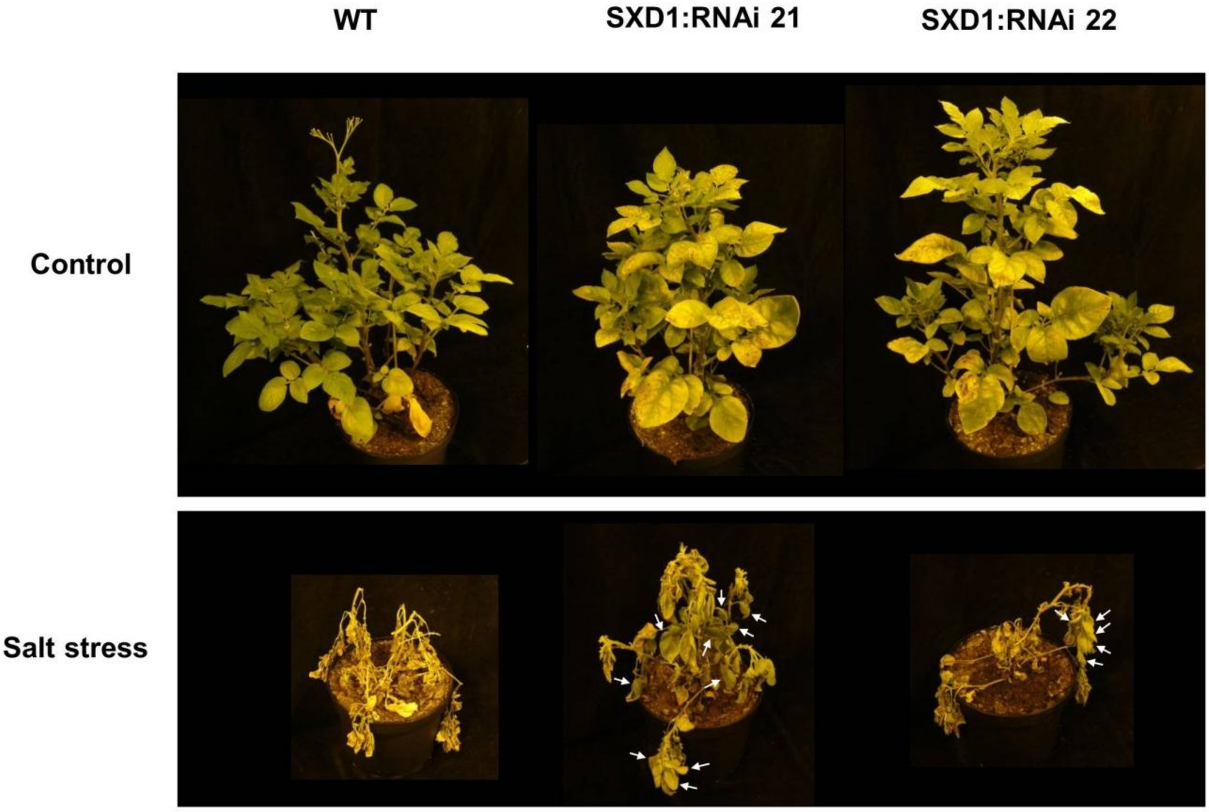

Figure S5. Phenotype of 11-week-old tocopherol-deficient potato plants compared to wild type after the end of the salt stress treatment. Pictures represent wild type plants (left images), SXD:RNAi-21 line (middle images) and SXD:RNAi-22 line (right images), and were taken 42 days after the onset of treatments (control plants, upper images; salt stressed plants, lower images). While salt stressed control plants were already dead at that time point, salt stressed tocopherol-deficient retained some green leaves (white arrows), especially line SXD1:RNAi-21.

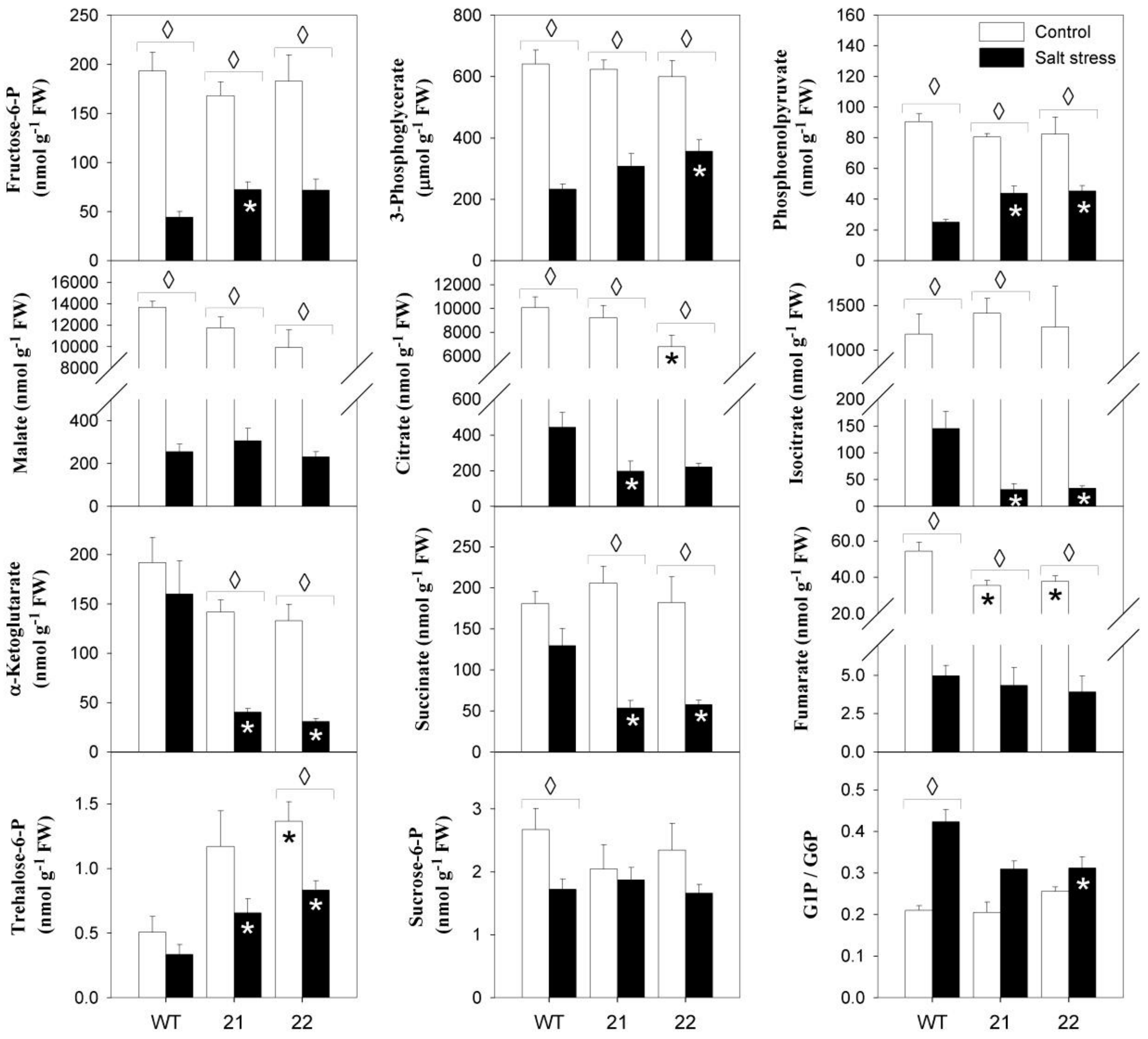

Figure S6. Contents of intermediates of central carbon metabolism in source leaves of SXD1:RNAi transgenic potato plants upon salt exposure. Measurements were performed in middle leaves (leaf 8) after 19 days treatment (salt stress, black bars; control, white bars). G1P/G6P, glucose-1-P to glucose-6-P ratio. Data represent the means ± SE of 4-5 individual leaves. Data were analyzed by *t*-test; significant differences between the transgenic lines and the wild type within a treatment are indicated by a black asterisk (control treatment) or a white asterisk (stress treatment), while diamonds indicate significant differences between control and salt stress within a genotype ($P < 0.05$).

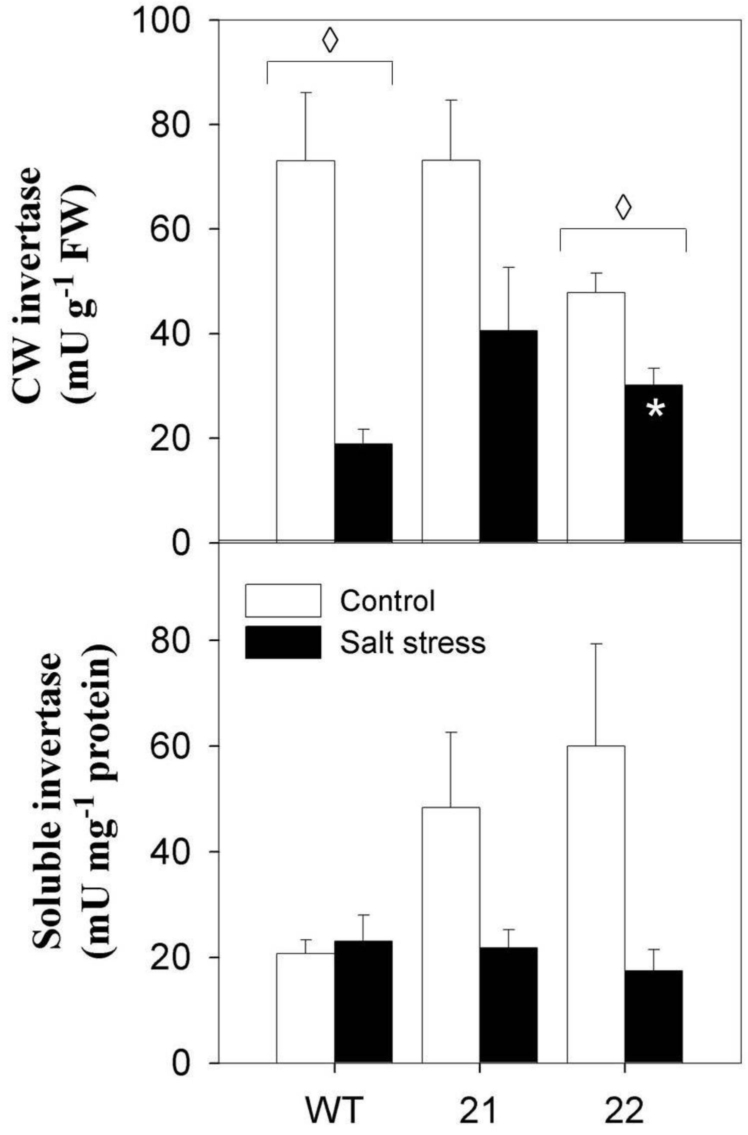

Figure S7. Soluble and cell-wall bound invertase activity in *StSXD1*-silenced potato plants after 19 days of salt stress. Enzymatic activity of cell wall (CW) invertase (top) and soluble invertase (bottom) was determined in middle leaves (leaf 8). Black bars, salt stress treatment; white bars, control treatment. Data represent the means ± SE of 4 individual plants. Data were analyzed by *t*-test; significant differences between the transgenic lines and the wild type within a treatment are indicated by a black asterisk (control treatment) or a white asterisk (stress treatment), while diamonds indicate significant differences between control and salt stress within a genotype ($P < 0.05$).

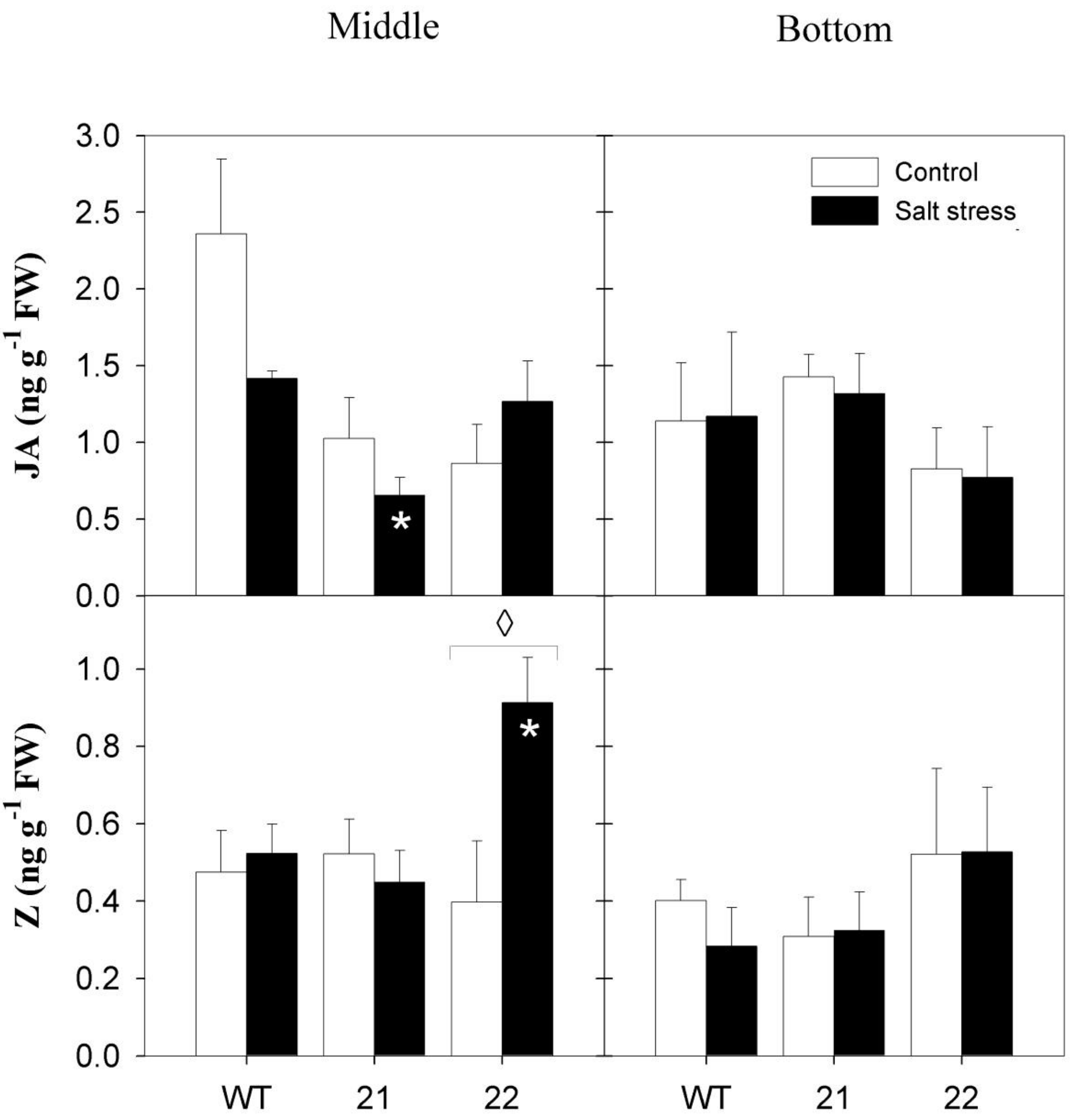

Figure S8. Phytohormone contents in source leaves of tocopherol-deficient potato plants exposed to 19 days salt treatment. Leaf content of JA (jasmonic acid) and of the cytokinin Z (zeatin) are shown from top to bottom. Left panels represent middle leaves (leaf 8); right panels represent bottom leaves (leaf 11). Black bars, salt stress treatment; white bars, control treatment. Data represent the means ± SE of 4 individual plants. Data were analyzed by *t*-test; significant differences between the transgenic lines and the wild type within a treatment are indicated by a black asterisk (control treatment) or a white asterisk (stress treatment), while diamonds indicate significant differences between control and salt stress within a genotype ($P < 0.05$).
